# Metal-Free and Degradable Photocatalyst for Water Decontamination: An Innovative Application for High-Sulfur Content Polymers

**DOI:** 10.1021/acsomega.5c04577

**Published:** 2025-10-17

**Authors:** Vinicius Diniz, Brenda Resendiz-Diaz, Colin R. Crick

**Affiliations:** † School of Engineering and Materials Sciences, 4617 Queen Mary University of London, London E1 4NS, U.K.; ‡ Institute of Chemistry, University of Campinas, Campinas 13083-970, Brazil

## Abstract

This study explores high-sulfur content polymers as metal-free photocatalysts for water purification. Photocatalytic activity was strongly influenced by the chemical structure of the cross-linker; polymers with fewer unsaturations showed higher performance, while siloxane groups reduced activity by 81.3% by hindering hydroxyl radical generation. Increased sulfur content further improves photocatalytic activity, underscoring sulfur’s role in contaminant degradation. The polymers achieved >80% removal of dyes and emerging contaminants, maintaining effectiveness in tap water, which demonstrates practical applicability. Reusability varied with cross-linker type, with 1,3-diisopropenylbenene (DIB)-based polymers showing lower durability than the 2,4,6,8-tetramethyl-2,4,6,8-tetravinylcyclotetrasiloxane-based polymers. Importantly, DIB polymers underwent UV-induced oxidation and mineralization, reducing environmental persistence and minimizing secondary pollution risks. Overall, these high-sulfur polymers represent a promising, environmentally friendly alternative to conventional metal-based photocatalysts for sustainable water purification and treatment.

## Graphical abstract

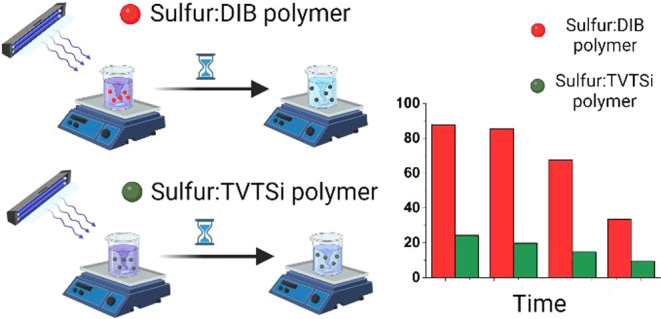

## 1 Introduction

Water scarcity has become a critical global issue, exacerbated by population growth, industrialization, and climate change. Global freshwater use has increased by 121% over the past five decades, while renewable freshwater resources have declined by 52.1%.^1^ Currently, 3.6 billion people live in countries experiencing water scarcity for at least one month annually,^2^ and unsafe water remains a major health and environmental problem, contributing significantly to the global burden of disease.^3^ These alarming statistics underscore the urgent need for innovative and sustainable solutions to protect and preserve water supplies. At the same time, industrial pollution is a major contributor to water contamination, with synthetic dyes emerging as a major environmental concern.^4,5^ Dyes are widely used in various industries, such as textiles, food processing, and pharmaceuticals.^6^ Their release into water bodies poses significant ecological risks, as many dyes are resistant to biodegradation and can persist in the environment for long periods.^7,8^ Furthermore, these contaminants can interfere with aquatic ecosystems and present health risks to humans due to their potential toxicity and carcinogenicity.^6,9^ Traditional water treatment technologies, while effective for some pollutants, often struggle to adequately remove these complex organic molecules from wastewater.^5,10^

Photocatalysis has emerged as a promising approach for the degradation of organic pollutants, including dyes, in water.^5,11–13^ Photocatalytic processes rely on light energy to drive chemical reactions that break down harmful substances into less toxic or inert compounds.^14^ However, many conventional photocatalysts are metal-based materials, such as titanium dioxide (TiO_2_), zinc oxide (ZnO), or bismuth-based oxides.^12,13,15–18^ While effective, these materials present several challenges, including high costs, limited availability of certain metals, and potential environmental toxicity.^19,20^ Moreover, the recovery and reuse of metal-based photocatalysts can be complex, leading to secondary pollution.

In response to these challenges, the development of metal-free photocatalysts has garnered significant attention as a response to the challenges posed by conventional metal-based catalysts.^1,21–23^ Since 2009, approximately 2282 articles on metal-free photocatalysts have been published (Scopus search: metal-free photocatalyst), yet only around 6% have explored their use for water treatment. Crick et al.^1,24^ have recently proposed the use of sulfur polymers, synthesized via inverse vulcanization, for degrading organic contaminants from water. First introduced by Pyun et al.^25^ to stabilize polysulfide chains with unsaturated cross-linking agents, inverse vulcanization results in materials with distinct chemical and physical properties suitable for advanced catalysis. This method is both solvent-free and sustainable,^26^ offering an efficient and straightforward approach to synthesize metal-free photocatalysts. Moreover, sulfur, an abundant and inexpensive byproduct of the petrochemical industry, can be used in large quantities, reducing the dependence on scarce or hazardous metals.^25,26^ When irradiated, α-sulfur generates electron–hole pairs similar to TiO_2_, functioning as a semiconductor, which produces ^•^OH radicals capable of degrading organic contaminants.^27,28^ Therefore, as sulfur polymers can be tailored for specific applications,^1,24,29–36^ these sulfur-based metal-free photocatalysts are promising alternatives for water decontamination,

This study pioneers the exploration of sulfur-rich, metal-free polymers as photocatalysts for removing industrial dyes and emerging contaminants from water, uniquely coupling high photocatalytic performance with the valorization of surplus elemental sulfur, thereby offering a sustainable and cost-effective alternative. Specifically, this study aims to (i) investigate the impact of cross-linking agents with varying degrees of unsaturation on photocatalytic activity; (ii) assess the effects of initial dye concentration, polymer loading, temperature, sulfur content, pH, ionic strength, and water matrix on polymer performance; (iii) evaluate the ability of the polymer to degrade emerging contaminants, using caffeine as a model; and (iv) examine the reusability of the sulfur polymer over multiple cycles of irradiation. We anticipate that the sulfur polymers have shown great potential to degrade organic contaminants, making them a promising alternative to metal catalysts. Additionally, unlike metal catalysts, sulfur polymers do not require complex separation steps for removal from water, as they pose no risks to aquatic ecosystems^26,37,38^ and exhibit degradability properties that can be tailored through the synthetic process.

## 2 Materials and Methods

### 2.1 Chemicals

1,3-diisopropenylbenzene (DIB) (stabilized with TBC, ≥97%) and elemental sulfur (S8, ≥99%), were purchased from Tokyo Chemical Industry (U.K.). Caffeine (99.0%), linseed oil (Lin), (*S*)-(−)-perillyl alcohol (PER) (≥95%), methylene blue hydrate (≥97.0%), 2,4,6,8-tetramethyl-2,4,6,8-tetravinylcyclotetrasiloxane (TVTSi) (≥95.0%), and Zn­(DTC)_2_ (97.0%) were purchased from Merck Ltd. (U.K.). Chloroform (99.8%), hydrochloric acid (37%), and sodium hydroxide (NaOH, reagent grade) were purchased from Fischer Scientific Ltd. (U.K.). Deuterated chloroform (99.8%) was purchased from Cambridge Isotopes Laboratories Ltd. (U.K.).

### 2.2 Polymer Synthesis

The polymers were synthesized according to the methodology described by Crick et al.^1,29,30^ with some modifications on the mixing step. Briefly, sulfur and cross-linker were weighed into a 14 mL vial at varied ratios. The vial was sealed with parafilm, vortexed for 60 s, and sonicated for 15 min. The mixture was then transferred to a hot plate with magnetic stirring and heated at 160 °C until a color change was observed. The prepolymers were then poured into silicone molds and cured at 140 °C overnight. After cooling, the polymers were ground using a mortar and pestle and used without further modification.

#### 2.3.1 Characterization

Scanning Electron Microscopy (SEM) imaging was performed using an FEI Inspect F system with an operational acceleration voltage of 10–20 kV. To enhance electrical conductivity within the SEM, samples were sputter-coated with a thin layer (≈10 nm) of gold using an Automatic Sputter Coater. Fourier Transform Infrared (FTIR) spectra were recorded using a Bruker Tensor 27 instrument over the wavenumber range of 500 to 4000 cm^–1^. Nuclear Magnetic Resonance (^1^H NMR) analysis was performed using a Bruker Advance DRX (400 MHz) spectrometer, with deuterated chloroform as the solvent and tetramethylsilane as the internal standard. Differential Scanning Calorimetry (DSC) measurements were carried out using a TA Instruments Discovery Series DSC 25. A heat–cool-heat method was employed, with heating and cooling rates set at 10 °C min^–1^ under a nitrogen atmosphere, spanning from −90 to 120 °C. Powder X-ray diffraction (PXRD) patterns were collected in reflection mode using a Panalytical X’Pert PRO MPD equipped with a high-throughput screening XYZ stage, X-ray focusing mirror, and PIXcel detector. Cu Kα radiation (λ = 1.5406 Å) was utilized, and data were collected over a range of 5–70° (step ≈ 0.03°) using loose powder samples on thin Mylar film within aluminum well plates. Thermogravimetric analysis (TGA) was conducted under an inert atmosphere on a TA Instruments TGA 5500. Heating was carried out at a heating rate of 10 °C min^–1^, from room temperature to 600 °C. The point of zero charge (pH_PZC_) was determined according to Diniz et al.^39^ Briefly, a solution of 0.01 mol L^–1^ NaCl was divided equally into five 10 mL glass containers. The pH of each container was adjusted to a value between 2 and 12. Subsequently, 20 mg of polymer was added to each container, and the mixtures were roller-mixed at room temperature for 48 h to measure the final pH. Diffuse reflectance spectroscopy (DRS) measurements were carried out in a PerkinElmer Lambda 950 UV–vis spectrophotometer. For that, the polymers were hot-pressed (Collin P3100 E Hot Press) with a pressure of 20 bar at 140 °C for 3 min to produce a flat sheet.

### 2.3 Photodegradation Studies

The photocatalytic activity of the polymers was evaluated using a 25 W UV–C lamp (254 nm, 25 mW/cm^2^) positioned orthogonally (5 cm) to a 200 mL beaker containing 100 mL of dye solution. The system was shielded with aluminum foil to enhance photocatalytic efficiency and prevent direct exposure to UV light, ensuring safety during the experiment. The initial dye concentration ranged from 2.5 to 15 mg/L, and polymer loading varied from 0.025 to 0.400 g. The effects of initial pH (3–11), NaCl concentration (0, 1, and 10 g/L), and temperature (25–55 °C) were also investigated using the same setup. Methylene blue stock solution (1 g/L) was prepared in double-distilled water, with further dilutions made using the same water. The total removal efficiency was calculated using eq 1

1 $$R=\frac{\left(C_{0}-C_{i}\right)}{C_{0}}\times 100$$

Where *R* is the total removal efficiency (%), and *C*_0_ and *C_i_* are the initial dye concentration and dye concentration (mg/L) at time *i* (min), respectively.

Residual methylene blue concentration was determined using a PerkinElmer Lambda 35 UV–vis spectrophotometer at 665 nm, with a calibration curve ranging from 0.5 to 20 mg/L. Samples were separated from the solution using a syringe and filtered through a 0.22 μm syringe filter. Kinetic data were fitted to a first-order kinetic model as previously described by our group.^17^

Additional experiments using tap water obtained from Queen Mary University of London’s laboratory and caffeine as a contaminant were performed under the same conditions. Tap water was selected as a representative matrix of real-world conditions, as its quality closely resembles that of water typically entering point-of-use catalytic and disinfection units in advanced water treatment systems.^40,41^ Additionally, caffeine was chosen as a model emerging contaminant due to its frequent detection in treated effluents and its relevance as a common marker of anthropogenic pollution.^39^ The caffeine stock solution was prepared in methanol, and subsequent dilutions were made in double-distilled water. Reusability tests were conducted over 8 h (4 cycles), with the solution spiked every 2 h with 0.8 mL of 100 mg/L methylene blue solution. All the experiments were performed in duplicate.

## 3 Results and Discussion

### 3.1 Polymers Synthesis

Inverse vulcanization transforms elemental sulfur into high-performance polymeric materials, using sulfur as the primary component and cross-linking it with small amounts of organic compounds^25^ (eq 2). The process involves heating sulfur above 159 °C, initiating S–S bond homolysis and ring-opening reactions that form reactive polysulfide chains. These chains are stabilized by adding unsaturated cross-linking agents, which trap the sulfur radicals and create a stable polymer network. In this work, four different cross-linkers were employed to stabilize the polysulfide chains: DIB (molecular weight (MW): 158.2 g/mol), PER (MW: 152.2 g/mol), TVTSi (MW: 344.7 g/mol), and linseed oil (MW: 282.5 g/mol) (Figure 1).

2 $$nS_{8}+m\left(CH=R\right)\underset{\mathrm{heat}}{\to }polymer\left(-R-C-S_{a}-C-R-\right)$$

Where *n* and *m* are the mass of sulfur and cross-linker, respectively, *a* is the length of the sulfur chain, and *R* is the cross-linker structure.

**Figure 1:**
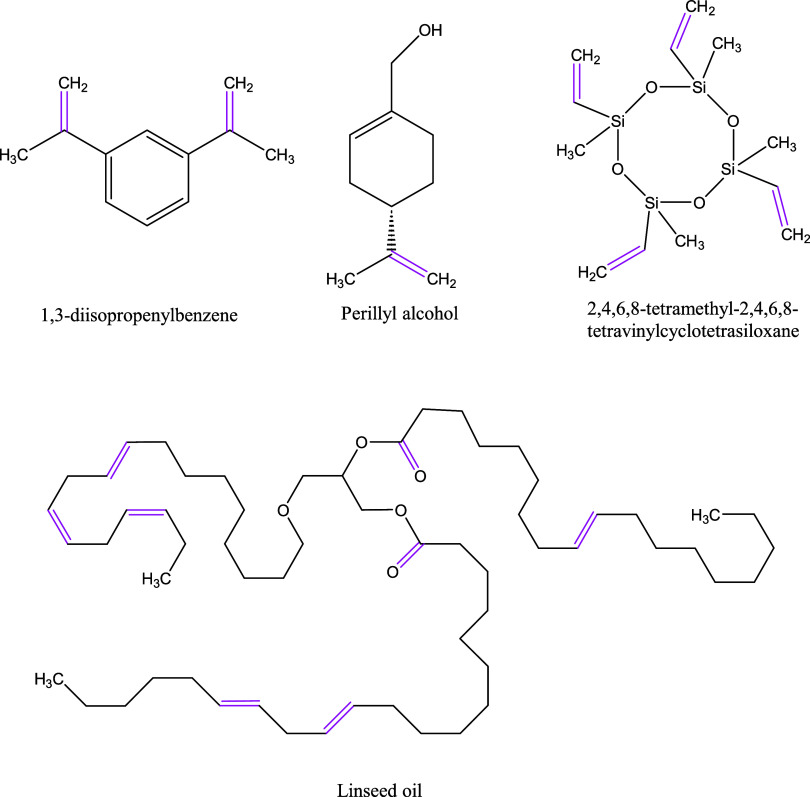
Chemical structure of the cross-linking agent used to synthesize the polymers. Reactive sites are highlighted in purple.

The successful polymerization, including the total or partial consumption of the cross-linker’s reactive sites, was confirmed through comparative analysis of the ^1^H NMR spectra of both the cross-linkers (see Figures S1–S4) and the resulting polymers (see Figures S5–S8). The ^1^H NMR spectra clearly showed the consumption of the reactive sites in the cross-linker structure, indicating the formation of a sulfur-cross-linker network. FTIR analysis of the polymers (Figure S9) further validated the reaction by highlighting characteristic bond changes associated with the polymerization process. Additionally, FTIR analysis revealed the appearance of C–S bonds within the polymers.

The XRD patterns of elemental sulfur (JCPDS card No. 08–0247) and sulfur polymer are presented in Figure 2. Elemental sulfur exhibited sharp, well-defined diffraction peaks, characteristic of its crystalline structure.^42^ In contrast, sulfur polymers displayed broad diffraction peaks, indicating their amorphous nature. However, for both the TVTSi and Lin polymers, a small peak at 2⊖ = 23°, corresponding to the *F*_ddd_ orthorhombic structure of sulfur^43^ was observed in the final polymer. This suggests that despite successful polymerization, a fraction of crystalline sulfur remained unreacted.

**Figure 2:**
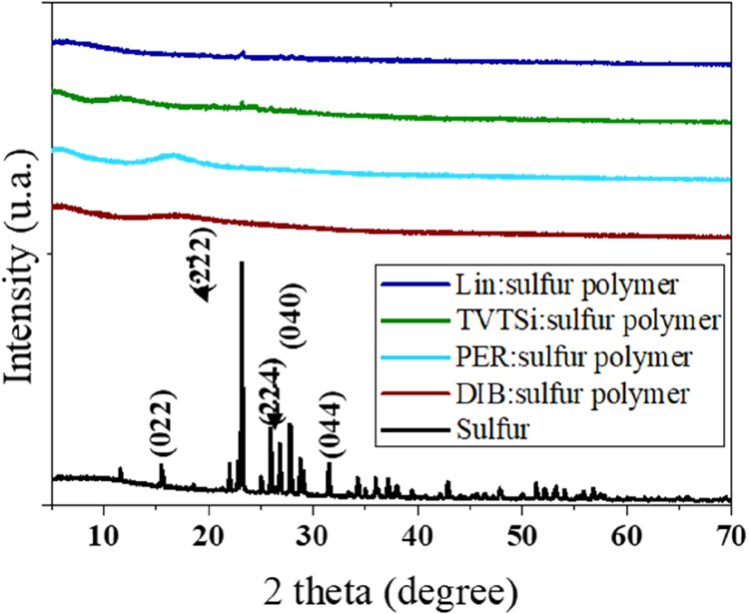
XRD analysis of the sulfur polymer synthesized with various cross-linkers. DIB: 1,3-diisopropenylbenzene; PER: Perillyl alcohol; TVTSi: 2,4,6,8-tetramethyl-2,4,6,8-tetravinylcyclotetrasiloxane; and Lin: linseed oil.

DSC analysis further confirmed the extent of sulfur consumption and provided the glass transition temperatures (*T*_g_) of the polymers (Figure 3). Notably, the *T*_g_ was found to correlate with the cross-linkers’ MW. The DIB polymer exhibited the highest *T*_g_ of 35.3 °C, while the TVTSi polymer had the lowest *T*_g_ at −29.4 °C. Both Lin polymer and TVTSi polymer showed *T*_g_ values below room temperature, indicating rubber-like behavior, which suggests the formation of longer sulfur–sulfur chains in these polymers compared to those formed in the DIB polymer and PER polymer.

**Figure 3:**
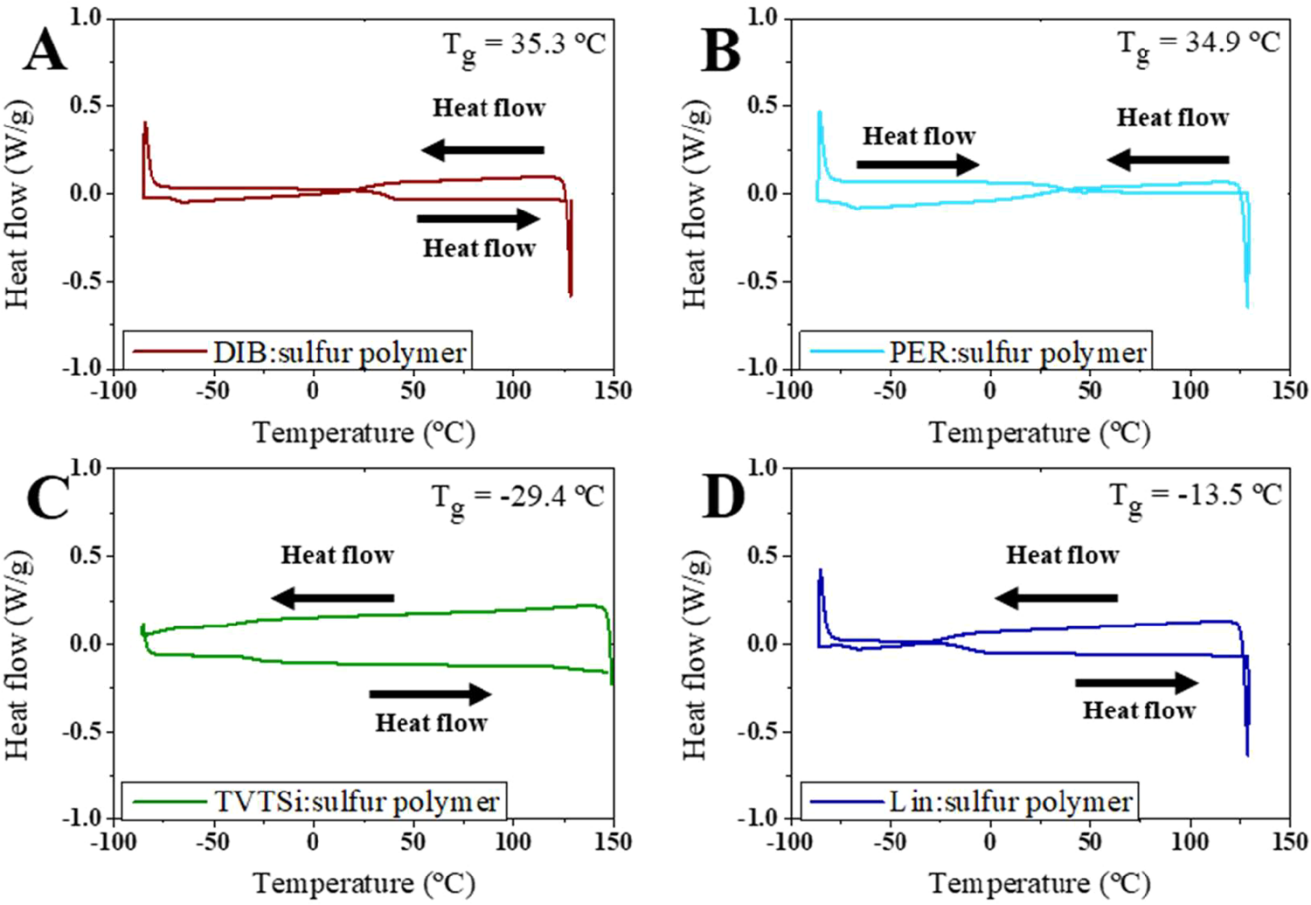
DSC thermograms of the sulfur polymer synthesized with various cross-linkers. (A) DIB: 1,3-diisopropenylbenzene; (B) PER: Perillyl alcohol; (C) TVTSi: 2,4,6,8-tetramethyl-2,4,6,8-tetravinylcyclotetrasiloxane; and (D) Lin: linseed oil. *T*_g_: glass transition temperature.

In order to investigate the thermal properties of the polymers, TGA analysis was performed (Figure 4). The analysis showed that, irrespective of the cross-linker, a similar residual mass (10–20%) was obtained at 600 °C. However, the decomposition patterns were different among the polymers. Both DIB and PER polymers exhibited a single mass loss <255 °C, suggesting the formation of the short sulfur–sulfur chains.^24^ On the other hand, both TVTSi and Lin polymers exhibited a second mass loss at >300 °C, which is attributed to the formation of longer-chain polysulfides. These observations corroborate the results observed in the DSC analysis, further confirming the formation of shorter chains in the DIB and PER polymers, while longer chains were formed in the TVTSi and Lin polymers. Finally, SEM was conducted to investigate the morphology of sulfur polymers after crushing them. All the polymers displayed a similar morphology, characterized by irregular, solid (nonporous) structures (Figure 4).

**Figure 4:**
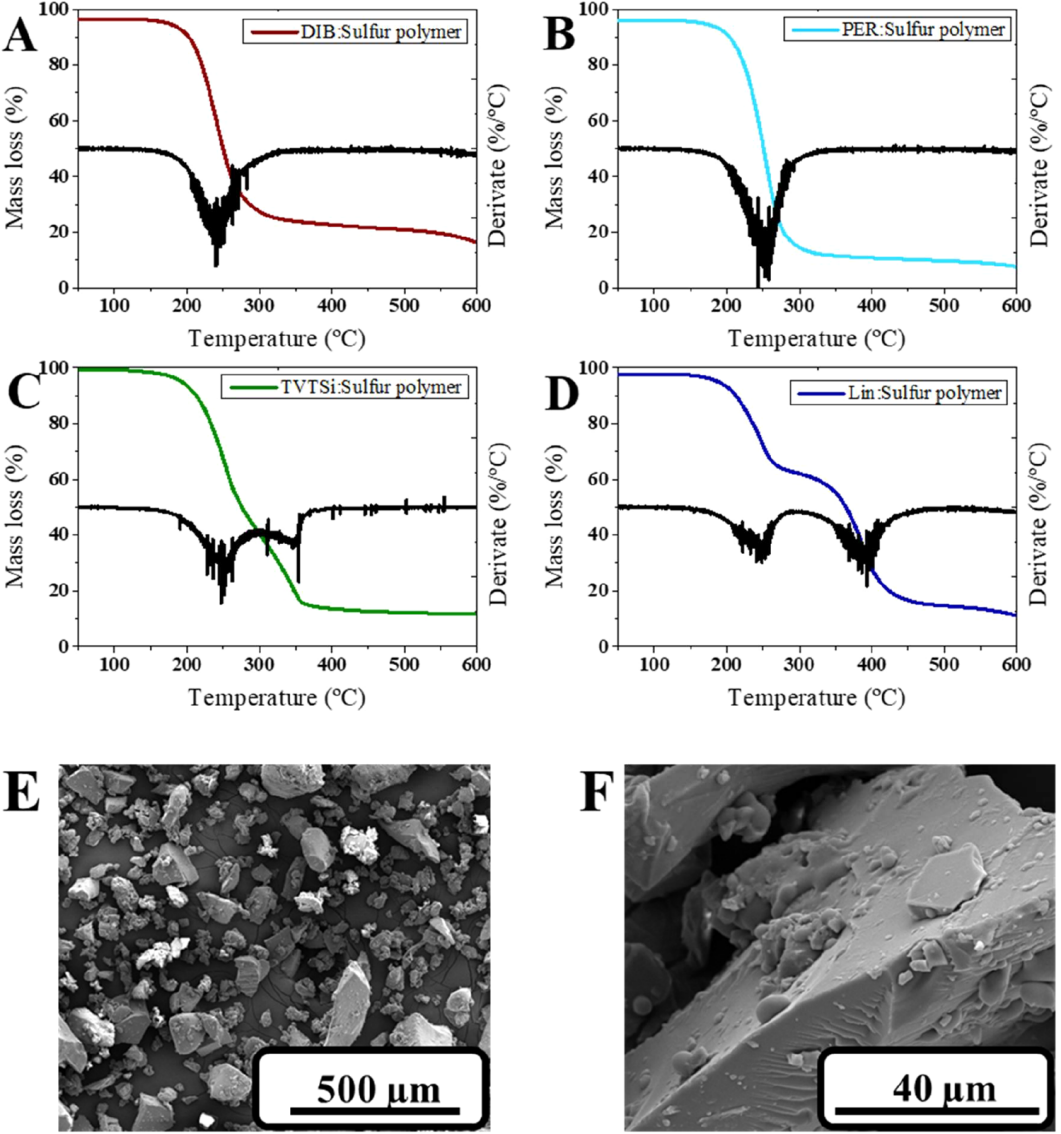
TGA analysis of the sulfur polymer synthesized with various cross-linkers (A) DIB: 1,3-diisopropenylbenzene; (B) PER: Perillyl alcohol; (C) TVTSi: 2,4,6,8-tetramethyl-2,4,6,8-tetravinylcyclotetrasiloxane; and (D) Lin: linseed oil. (E) and (F) SEM micrographs of DIB: sulfur polymer.

DRS was used to estimate the optical band gap of the synthesized polymers. The DIB/sulfur polymer exhibited a band gap of 2.08 eV (Figure S10), corresponding to light absorption up to approximately 550 nm. DRS analysis was also performed for the other polymers; however, due to their intense dark coloration, they exhibited minimal reflectance across the entire measured spectrum (800–190 nm), preventing the acquisition of reliable DRS spectra.

### 3.2 Photodegradation Performance of Sulfur Polymers: Effect of Cross-Linker

Sulfur-doped materials are well-known for their enhanced photocatalytic activity compared to their nondoped counterparts,^28,42,44,45^ positioning sulfur as a promising alternative for the development of alternative photocatalysts. Since the discovery of inverse vulcanization, limited studies have investigated the photocatalytic activity of these polymers. Most of the existing research has focused on their use to enhance the self-cleaning properties of superhydrophobic surfaces.^24,46^ In this study, the photocatalytic activity of sulfur polymers was investigated for breaking down organic contaminants in aqueous media.

The results demonstrated that the choice of cross-linker directly affected the photocatalytic activity of the polymer, with the total dye removal following the order of PER > DIB > Lin > TVTSi (Figure 5). However, it is noteworthy that for the experiments with TVTSi and Lin polymers, a higher polymer loading (0.500 vs 0.200 g) was used due to the lower photocatalytic activity of these polymers. To account for these differences, the normalized removal (also known as removal capacity) was calculated using eq 3 and is shown in Figure S10

3 $$q=\frac{\left(C_{0}-C_{i}\right)\times V}{m}$$

Where *q* is the removal capacity (mg/g), *C*_0_ and *C_i_* are the initial dye concentration and dye concentration at time *I*, respectively, *V* is the volume (100 mL), and *m* is the mass of the polymer.

**Figure 5:**
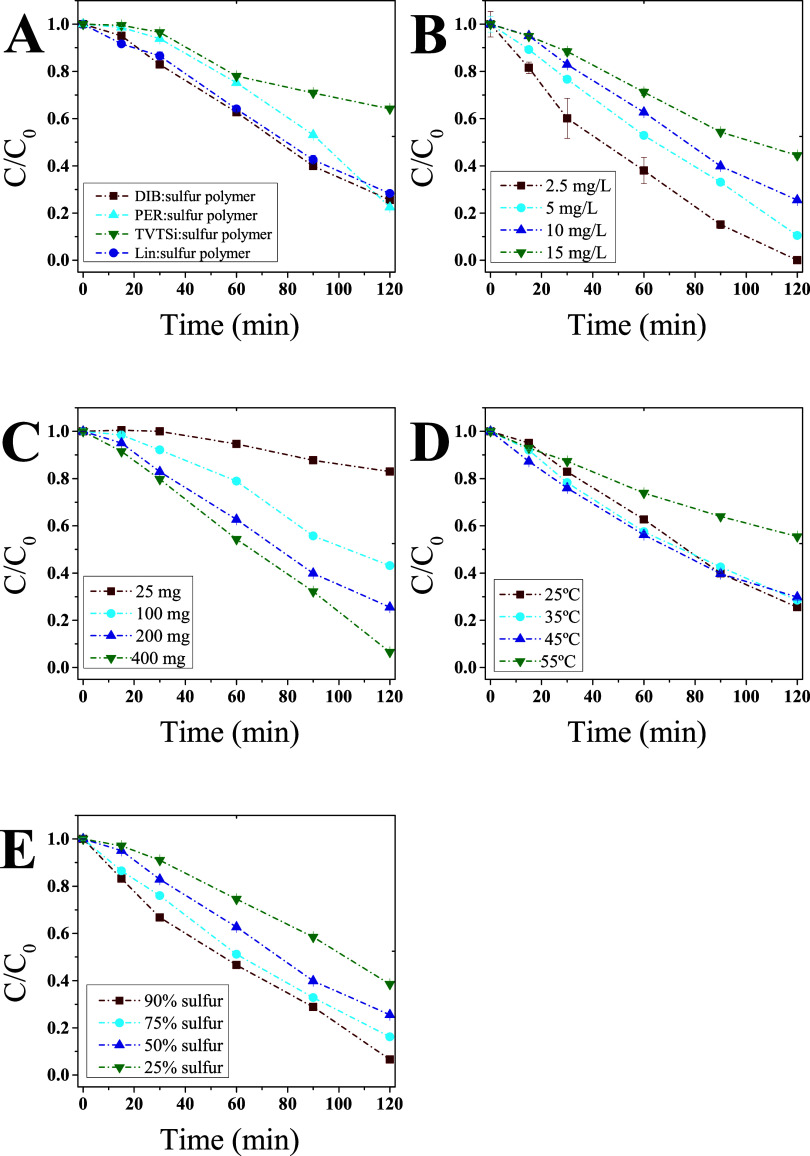
(A) Influence of the cross-linker on the photocatalytic activity of sulfur polymers. (B) Effect of initial dye concentration on the photocatalytic performance of DIB:sulfur polymer (C) Effect of DIB/sulfur polymer loading on dye degradation. (D) Effect of temperature on dye degradation by DIB:sulfur polymer. (E) Influence of sulfur content on dye degradation by DIB:sulfur polymer. DIB: 1,3-diisopropenylbenzene; PER: Perillyl alcohol; TVTSi: 2,4,6,8-tetramethyl-2,4,6,8-tetravinylcyclotetrasiloxane; and Lin: linseed oil.

After normalization, *q* values followed the order: PER (*q* = 4.3 mg/g) > DIB (*q* = 3.8 mg/g) > Lin (*q* = 1.9 mg/g) > TVTSi (*q* = 0.8 mg/g) (Table 1). Although the photocatalytic mechanism of these sulfur polymers is not fully understood, it is known that it derives from the photocatalytic properties of sulfur.^1,34,46^ In the same way, there is a lack of studies investigating the impact of cross-linkers on these polymers. Berk et al.^34^ reported higher photocatalytic activity for the polymer synthesized with oleic acid when compared to the polymers synthesized with linoleic and linolenic acids. While the authors did not explore these results further, their results suggest a direct correlation between the number of reactive sites in the cross-linkers and the photocatalytic activity, with a higher number of reactive sites leading to reduced activity. Consistent with those findings, both cross-linkers with the lowest number of reactive sites (DIB and PER) (see Figure 1) resulted in the polymers with the highest photocatalytic activity. On the other hand, while TVTSi has fewer reactive sites compared to Lin, the latter exhibited higher photocatalytic activity, indicating that other factors also influence the photocatalytic activity. The siloxane groups (Si–O–Si) in the TVTSi cross-linker are known to form a class of highly flexible backbones that hinder the interaction with hydroxyl radicals,^47^ potentially reducing the photocatalytic activity of TVTSi-based polymers. Taken together, these findings highlight that cross-linker chemistry provides a powerful means to tune photocatalytic performance, influencing both activity and stability. By selecting cross-linkers with different reactive site densities and backbone flexibility, it is possible to balance efficiency, UV stability, and radical accessibility, establishing a rational approach for designing sulfur polymers tailored to specific water treatment needs.

**Table 1:** Composition of the Synthesized Polymers and Their Efficiency in Degrading Methylene Blue (10 mg/L MB in 100 mL Solution at 25 °C)

cross-linker	sulfur content (%)	mass of polymer (mg)	*q* (mg/g)	removal (%)	*k* (1/min)
DIB	25	200	3.2	61.5	0.006
DIB	50	200	3.8	74.5	0.009
DIB	75	200	4.4	83.8	0.120
DIB	90	200	5.4	93.4	0.145
PER	50	200	4.3	77.4	0.007
Lin	50	500	1.9	71.7	0.009
TVTSi	50	500	0.8	39.5	0.003

Although PER (*k* = 0.007 min^–1^) had resulted in the polymers with the highest *q* values, DIB (*k* = 0.009 min^–1^) polymers were chosen for further experiments as they exhibited the highest removal rate (*k*) (determined using the first-order kinetics model (see Figure S11)). Higher removal rates are particularly important for real-world applications as rapid contaminant degradation is essential for the efficiency and scalability of water purification systems, reducing treatment times and energy consumption.

### 3.3 Factors Affecting Photocatalytic Activity

Photodegradation of organic contaminants is recognized to be a dynamic process influenced by numerous factors, particularly initial concentration, photocatalyst loading, temperature, pH, and salinity.^12,48^ As sulfur polymers are being explored for the first time as alternative metal-free photocatalysts for water purification, this work investigated not only these factors but also the effect of sulfur content in the polymers, as it is hypothesized that sulfur plays a key role in the photocatalytic activity of these materials.^1,34^

#### 3.3.1 Effect of Initial Concentration

The influence of the initial concentration of methylene blue on the efficiency of DIB:sulfur polymers was investigated using initial concentrations varying from 2.5 to 15 mg/L (Figure 5). For the lowest concentration investigated (2.5 mg/L), no residual dye was observed after 120 min (see Table S1 for more details). However, as the initial concentration increased, the removal efficiency decreased. For the highest concentration (15 mg/L), the removal efficiency was 55.6% after 120 min. It has been reported that the initial dye concentration has a significant impact on the photodegradation efficiency in heterogeneous photocatalytic systems, where several studies showed that increasing dye concentration results in reduced removal efficiency.^49–51^ This effect can be explained by the absorption of irradiation by the dye molecules, limiting the irradiation reaching the photocatalyst surface.^48^ As fewer reactive radicals are generated by the photocatalyst, the removal efficiency decreases at higher initial concentrations compared to lower ones, leading to reduced degradation performance.^52^

#### 3.3.2 Effect of Polymer Loading

To further understand the effect of polymer loading on the removal efficiency of methylene blue, photodegradation tests were conducted using varying amounts of polymer, ranging from 25 to 400 mg in 100 mL of a 20 mg/L dye solution (Figure 5). The results indicated that the removal efficiency was higher with higher polymer loading. For example, with 25 mg of polymer, the removal was 17.0%, whereas 400 mg of polymer achieved 93.5% removal after 120 min (see Table S2 for more details). This increase in removal can be attributed to several factors:^48^ (i) higher polymer loading facilitates the contact between the polymer and dye molecules, enhancing the probability of the dye molecule encountering the polymer surface; (ii) with a greater polymer mass, the concentration of radicals capable of degrading the dye molecules is significantly higher, leading to a more efficient and faster breakdown of the dye. However, while the removal (%) increases, the removal capacity decreases (Figure S12). This can be explained by the increased turbidity of the suspension in heterogeneous photocatalysis, where the dominant light scattering reduces the amount of light absorbed by the surface of the photocatalyst.^48^

#### 3.3.3 Effect of Temperature

To explore the effect of temperature on the photocatalytic activity of DIB:sulfur polymers, experiments were conducted at temperatures ranging from 25 to 55 °C (Figure 5 and see Table S3 for more details). The results revealed that temperatures up to 45 °C had no significant effect on methylene blue degradation, indicating that the polymers remain effective under most real-world conditions.^53^ Groeneveld et al.^48^ explain that the thermal energies involved in photodegradation are minimal compared to the photon energies absorbed, meaning temperature has little influence on degradation pathways. However, at the highest temperature (55 °C), a notable decrease in removal efficiency was observed. At high temperatures, the mobility of the dye molecules increases, which may limit their contact with the surface of the photocatalyst.^54^ In addition, at temperatures above the polymer’s *T*_g_, increased chain mobility allows free radicals within the polymer to react with each other more readily,^55^ potentially degrading the polymer itself rather than breaking down the dye molecules, thereby diminishing photocatalytic activity toward the contaminant. Further studies are needed to better understand the effect of *T*_g_ on the photocatalytic activity of sulfur polymers, as it has been observed to play a significant role in influencing their photocatalytic efficiency.

#### 3.3.4 Effect of Sulfur Content

As sulfur is hypothesized to play a major role in the photocatalytic activity of sulfur polymers, the effect of sulfur content on methylene blue degradation by DIB:sulfur polymers was also investigated. For that, polymers with 25%, 50%, 75%, and 90% sulfur were synthesized as previously described, and their removal efficiency was tested by adding 200 mg of polymer to 100 mL of 10 mg/L methylene blue solution. The results demonstrated a direct correlation between sulfur content and removal efficiency (Figure 5 and Table 1), corroborating previous literature findings.^1,34^ For example, the polymer containing 90% sulfur removed 93.4% of methylene blue after 120 min, while the polymer with only 25% sulfur achieved a lower removal rate of 61.5%.

α-sulfur crystals have been recognized as interesting photocatalysts for the degradation of organic contaminants.^28,56,57^ According to Liu et al.^56^ α-sulfur crystals are capable of generating hydroxyl radicals (^•^OH) from hydroxide ions due to the sufficiently high oxidation potential of their valence and conduction bands, enabling this reaction. However, pure α-sulfur suffers from rapid recombination of photogenerated electron–hole pairs, which limits its effectiveness for water purification.^58^ When heated to 250 °C, α-sulfur transforms into polymeric sulfur, forming long chains with radical end groups that enhance photocatalytic activity and reduce recombination rates.^28,59^ However, this polymeric sulfur is unstable and tends to depolymerize.^25^ Therefore, using unsaturated cross-linkers in inverse vulcanization is a viable strategy to harness the photocatalytic activity of polymeric sulfur, highlighting sulfur’s key role in the performance of DIB:sulfur polymers. These results emphasize that sulfur loading primarily governs the overall magnitude of photocatalytic activity, with higher sulfur content enhancing radical generation and removal efficiency. Thus, sulfur percentage acts as a key lever for tuning activity levels to match treatment requirements

#### 3.3.5 Effect of pH and Ionic Strength

Another key factor influencing photocatalyst efficiency is pH, as it directly affects both the surface charge of the material and the charge of redox-active groups.^48^ In the case of DIB:sulfur polymers, the degradation of methylene blue remained consistent across most of the tested pH range, except at pH 5, where the removal dropped to 32.2% (Figure 6). Given the methylene blue p*K*_a_ of 3.8, higher degradation is expected at pH values below this threshold, where the dye primarily exists in its cationic form (at pH 3, the fraction of MB^+^ is 0.863).^60^ On the other hand, sulfur-based photocatalysts generally show strong activity at higher pH values.^57^ These results support the hypothesis that the primary mechanism driving the photocatalytic activity of sulfur polymers involves the oxidation of the dye by hydroxyl radicals (eqs 4 to 6).

4 $$sulfur\;polymer+\mathrm{hv}\to e^{-}+h^{+}$$

5 OH−+h+→OH•

6 OH•+MB→degradationproducts

Where *h*v is the photon energy (irradiation), e^–^ is the electron of the conduction band, h*^+^* is the hole of the valence band, OH^–^ is the hydroxide ions, ^•^OH is the hydroxyl radicals, and MB is methylene blue.

**Figure 6:**
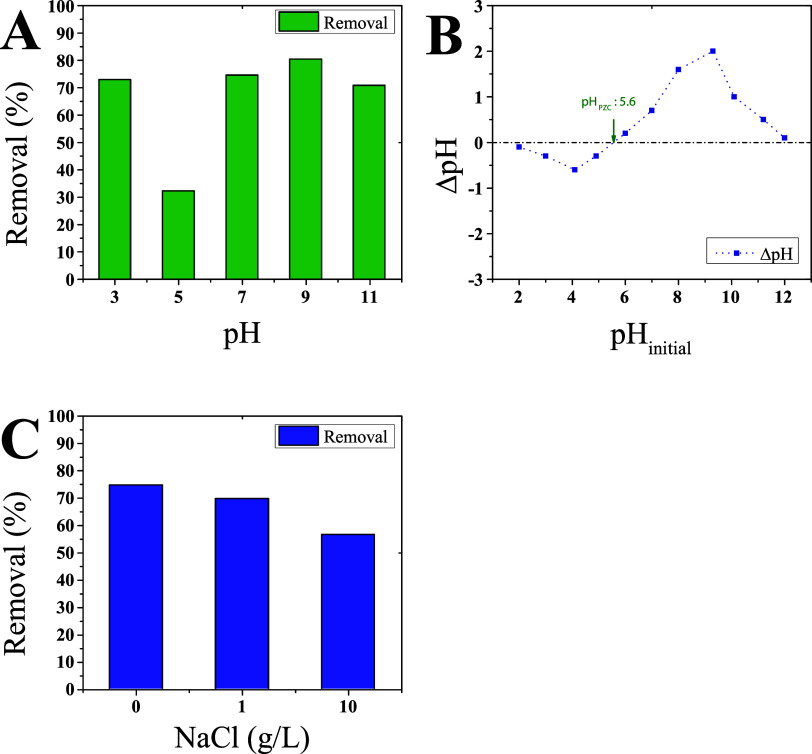
(A) Effect of pH on the degradation of dye by DIB:sulfur polymer. (B) pH_PZC_ pattern of DIB:sulfur polymer. (C) Effect of NaCl concentration on the degradation of dye by DIB:sulfur polymer. DIB: 1,3-diisopropenylbenzene.

At pH 5, the generation of hydroxyl radicals is limited, as water oxidation becomes the main radical formation pathway, and methylene blue is less prone to photodegradation.^60^ Additionally, since the sulfur polymers synthesized in this work are nonporous (Figure 4), adsorption plays a minimal role in the process. The pH_PZC_ of the polymer also appears to have little influence, as high removal rates were observed even under conditions where both the dye and the polymer are likely positively charged.

To further understand the factors influencing the photocatalytic activity of sulfur polymers, the effects on ionic strength were investigated through experiments with 0, 1, and 10 g/L of NaCl (Figure 6). Results revealed that higher NaCl concentration led to reduced removal efficiency (*i.e*., from 74.5% without NaCl to 56.4% at 10 g/L). This supports the hypothesis that hydroxyl radicals are the main oxidant species, as NaCl can scavenge both photogenerated holes and hydroxyl radicals^61^ (eqs 7 and 8). Although chloride radicals can also oxidize organic compounds, their lower redox potential makes them less effective compared to hydroxyl radicals,^62^ explaining the reduced performance at higher concentrations of NaCl.

7 Cl−+h+→Cl•

8 Cl−+OH•→OH−+Cl•

Where, Cl^–^ is the chloride ion, h^+^ is the hole of the valence band, ^•^Cl is the chloride radical, ^•^OH is the hydroxyl radical, and OH^–^ is the hydroxide ions.

It is noteworthy that trapping experiments of active species could serve as an alternative approach to further investigate the mechanism behind the photocatalytic activity of sulfur polymers. However, these polymers exhibit varied compatibility with hydroxyl radical scavengers (e.g., isopropanol^30^), which could affect the reliability of the results. Overall, photocatalytic performance appears to be governed by the relative amount of elemental sulfur, its degree of crystallinity, and the chemical nature of the organic cross-linker, highlighting the importance of polymer composition and structure in tuning activity.

#### 3.3.6 Effect of Water Matrix

The degradation of methylene blue was also assessed using tap water. Tap water presents a more complex matrix due to the presence of various ions and organic matter, which can influence the photocatalytic activity.^63^ Interestingly, a slightly higher removal was observed when the degradation was performed in tap water (78.7%) compared to deionized water (74.5%) (Figure S13). Despite the presence of inorganic salts in tap water that can act as hydroxyl radical scavengers,^12^ their concentrations are relatively low (Table S4), which minimizes their negative effect on the photocatalytic process. Additionally, the organic contaminants concentration in tap water (represented as total organic carbon) is 5 times lower than the initial dye concentration, thus having little impact on the removal of methylene blue. The presence of sulfate in tap water (54 mg/L) may also enhance photocatalytic activity by generating sulfate radicals,^28,64^ which could explain our findings. The results indicate that sulfur polymers are effective in tap water; however, further studies in more complex matrices, such as river water or treated effluent, are needed to fully assess their potential as metal-free photocatalysts. Nevertheless, this study highlights the viability of sulfur polymers for water treatment, even in environments with higher complexity than deionized water.

#### 3.3.7 Removal of Emerging Contaminants

Emerging contaminants are recognized as critical threats to human health and the sustainability of ecosystems, requiring efforts for their removal/breakdown from water matrices.^41,65^ Due to the large number of emerging contaminants, it is virtually impossible to assess the removal of all of them and therefore prioritization is a critical step when working with emerging contaminants.^40,66^ For this study, caffeine was selected as a model for emerging contaminants because it has been previously prioritized and is a well-known anthropogenic marker widely consumed by humans.^67^ Caffeine removal kinetics are shown in Figure S14, demonstrating a degradation pattern similar to that of methylene blue (*i.e*., higher polymer loading resulted in increased removal efficiency). However, the polymer showed a higher removal efficiency for caffeine compared to methylene blue (Figure 7). This can be attributed to the C–H bond between two nitrogen atoms in caffeine molecule, which is more easily oxidized by radicals, making caffeine more susceptible to photodegradation by hydroxyl radicals than methylene blue.^68^ Additionally, it is noteworthy that the caffeine experiment contained a residual 1% methanol from the stock solution, suggesting that the removal efficiency could be even higher in the absence of methanol. These findings indicate that sulfur polymers are also a promising alternative for the removal of emerging contaminants. Nevertheless, further experiments are needed to evaluate other classes, such as antimicrobials and pesticides, to fully assess their potential in diverse applications.

**Figure 7:**
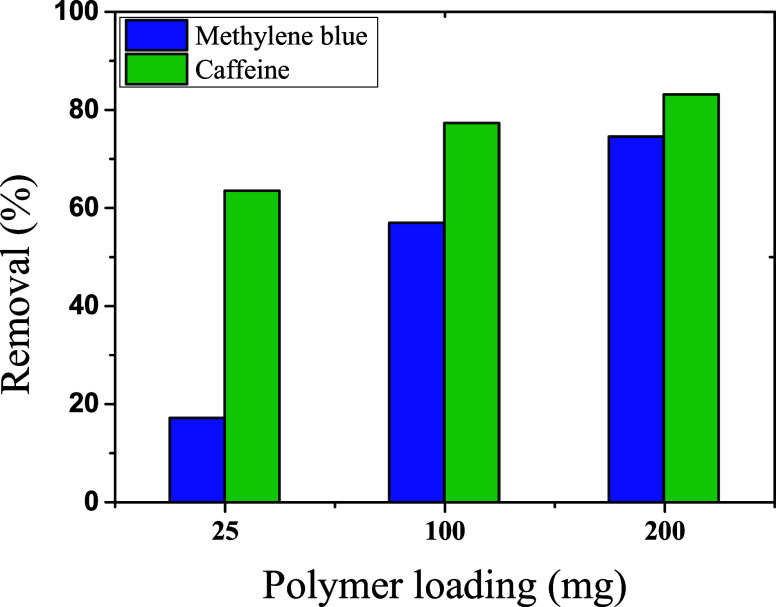
Removal efficiency of methylene blue and caffeine by 1,3-diisopropenylbenze:sulfur polymers.

#### 3.3.8 Performance Comparison with TiO_2_ Catalysts

The photocatalytic performance of the DIB:sulfur polymer was evaluated and compared to conventional metal-based catalysts such as TiO_2_ and its composites. Under UV–C irradiation (254 nm, 25 W), the DIB:sulfur polymer achieved 93.4% removal of a methylene blue (10 mg/L) in 120 min using a catalyst loading of 2000 mg/L. This performance is comparable to or even superior to several TiO_2_-based systems reported in the literature. For instance, 15% TiO_2_/activated carbon composites removed 98.3% of a more concentrated solution (160 mg/L) under 15 W UV in the same duration, whereas pure TiO_2_ achieved only 40–60% removal depending on the light intensity and experimental conditions.^69,70^ Moreover, under similar concentration conditions (10 mg/L), TiO_2_ at 200 mg/L achieved 80% removal in 60 min using a 24 W UV–C lamp.^71^ Although the DIB:sulfur polymer required a higher catalyst loading, it matched or exceeded removal efficiencies under comparable or milder UV conditions. Importantly, unlike TiO_2_, the DIB:sulfur polymer is metal-free, amorphous, and degradable, offering an environmentally benign and sustainable alternative for photocatalytic water purification. These findings highlight the potential of inverse vulcanized polymers as effective photocatalysts and justify further investigation into their mechanisms and scalability.

### 3.4 Reusability

The lifespan of photocatalysts is crucial for both sustainability and cost-effectiveness. As such, evaluating the reusability of photocatalysts over multiple cycles is a common approach to assess their long-term viability in water purification.^72^ Balancing photocatalytic activity with material degradability is a key challenge for sulfur polymers: while rapid polymer degradation shortens catalyst lifetime, it also reduces its environmental persistence and associated risks.

Here, the reusability of DIB:sulfur polymers was tested over four cycles, with the solution spiked with concentrated methylene blue solution (100 mg/L) every 120 min. The results showed that removal efficiency remained nearly constant during the first 240 min of degradation (Figure 8), after which it declined exponentially as described by eq 9, where *a* is 0.043 and *b* is 0.0057.

9 $$C/C_{0}=a\times e^{b\times \mathrm{time}}$$

Where *C* and *C*_0_ are the concentrations of dye at time *i* and 0 respectively, *a* is the initial value (or pre-exponential factor), and *b* is the rate coefficient associated with the loss of photocatalytic efficiency.

**Figure 8:**
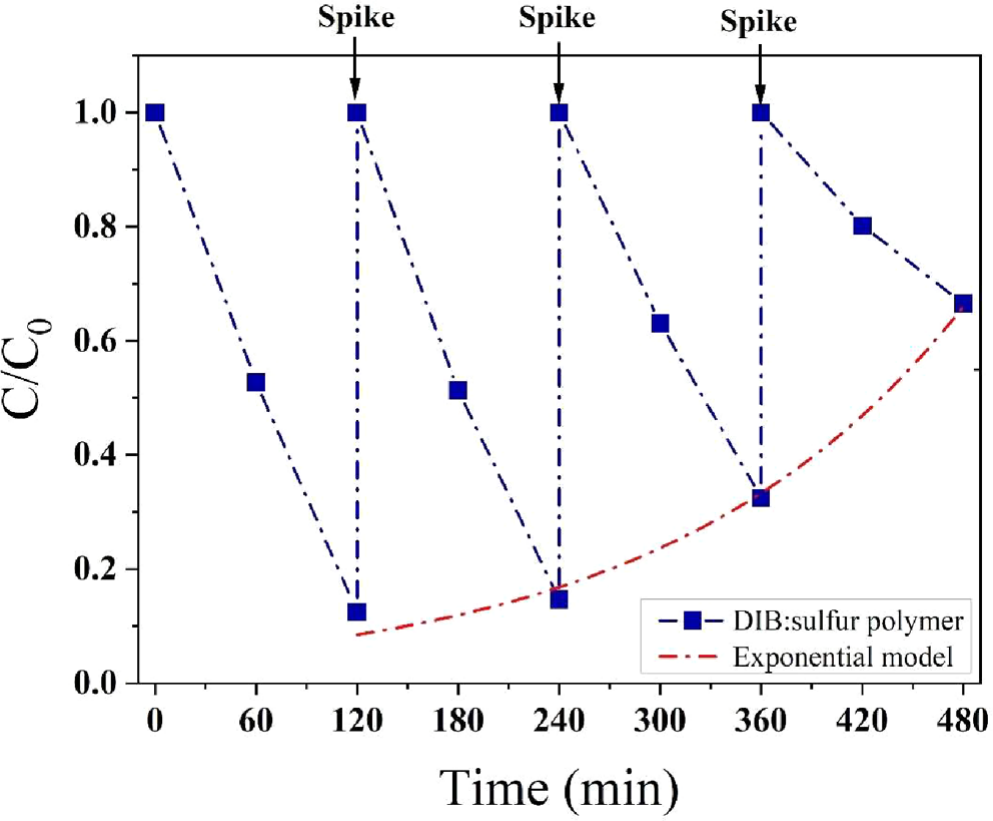
Reusability of 1,3-diisopropenylbenzene:sulfur polymer over four photodegradation cycles. The red dash-dotted line represents an exponential model used to predict the removal efficiency at the end of each cycle.

Metal photocatalysts often maintain high efficiency over several cycles,^73–75^ but their nanoscale dimensions make separation from water difficult, raising concerns about secondary pollution.^76^ Although strategies such as incorporating magnetic precursors facilitate separation,^77^ these approaches often involve complex synthetic procedures and the use of metal-based materials, which introduces additional environmental and cost-related concerns. In contrast, sulfur polymers may degrade to less harmful products under UV irradiation, potentially reducing secondary pollution risks. However, their inherent degradability must be considered carefully depending on the application.

To evaluate the degradability of DIB:sulfur polymers, their physicochemical properties were monitored throughout four cycles of methylene blue degradation. SEM analysis revealed that, after 8 h of irradiation, the polymer developed a more irregular surface morphology compared to the smooth surface in the nonirradiated polymers, indicating structural degradation and oxidation processes (Figure S15). This was further supported by the disappearance of key peaks in the FTIR spectra, specifically the 2860 cm^–1^ peak, corresponding to the C–H stretching vibration of aliphatic (saturated) hydrocarbons, and the 1510 cm^–1^ peak, associated with the C=C stretching vibration in the aromatic benzene ring of DIB (Figure 9). Concurrently, a new peak around 1680 cm^–1^, attributed to carbonyl (C=O) stretching, emerged and increased with irradiation time (Figure S16).

**Figure 9:**
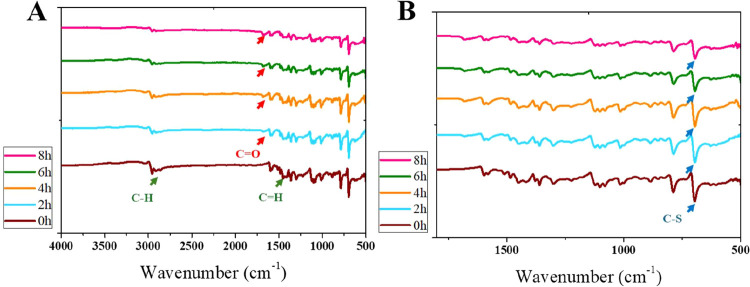
Zoomed-out (A) and zoomed-in (B) views of the FTIR spectra evolution of 1,3-diisopropenylbenzene:sulfur polymer exposed to UV-irradiation. Green arrows indicate peaks that disappear after time 0 h, while red arrows highlight the appearance of new peaks.

Elemental analysis further confirmed that the combined carbon, hydrogen, and sulfur content in the polymer decreased from 97.41% to 86.26% after 8 h of UV irradiation (Table 2). This reduction supports the hypothesis of polymer oxidation, suggesting that oxygen may have been incorporated into the polymer structure during the process. The decline in sulfur content suggests sulfur loss, possibly due to the cleavage of sulfur–sulfur bonds under UV light. This degradation may release sulfur species, such as sulfide ions (S^2–^) or sulfur oxides, which could account for the drop in sulfur levels. Additionally, this release correlates with the observed decrease in solution pH during irradiation (Figure S17), as the formation of acidic sulfur compounds contributes to acidification.^78^

**Table 2:** Elemental Analysis of 1,3-Diisopropenylbenezene/Sulfur Polymer before and after 8 h of UV Irradiation

	irradiation time (h)	
chemical element (%)	0	8
carbon	42.37	40.27
hydrogen	4.03	3.84
sulfur	51.01	42.15
total	97.41	86.26

Sulfur is considered nontoxic to aquatic biota,^26^ and sulfur polymers have demonstrated no toxicity to HepG2 and Huh7 liver cells.^37^ However, their toxicity after UV-induced degradation remains unassessed. Our findings suggest that the degradation of the sulfur polymer not only involves structural changes but also results in the release of sulfur species into the environment. Therefore, future studies must monitor the toxicity associated with polymer breakdown, particularly in the context of environmental exposure. Additionally, smoothing in the FTIR fingerprint region supports cross-linker mineralization and aromatic breakdown (Figure 9).

To further investigate UV-induced degradation, TGA analysis was performed. Interestingly, the results revealed an increase in thermal stability, as shown by a higher residual mass (Figure S18). Although oxidation typically reduces thermal stability by weakening chemical bonds, UV irradiation can also promote cross-linking. Jia et al.^79^ proposed photoinduced inverse vulcanization, with optimal synthesis occurring at 435 nm to minimize side reactions. In this study, a wavelength of 254 nm was used for photodegradation, likely promoted both degradation and additional cross-linking. This increased cross-linking is supported by the rise in decomposition temperature (Figure S18), and a higher *T*_g_ (Figure S19) after 8 h of irradiation, indicating the formation of a more robust polymer matrix.

#### 3.4.1 Tailoring Polymer Structure and Functionality

Although DIB:sulfur polymers exhibit limited lifespan and degrade after a few cycles, the tunable nature of sulfur polymers makes them highly versatile and adaptable for specific applications. Recently, Diniz et al.^24^ demonstrated that introducing siloxane groups via TVTSi can enhance UV stability. In this study, TVTSi:sulfur polymers exhibit reduced photocatalytic activity compared to other synthesized polymers. To further investigate whether this correlates with increased stability, reusability experiments were also performed.

As anticipated, the photocatalytic activity of TVTSi:sulfur polymers was lower than that of DIB, indicating reduced reactivity (Figure 10). On the other hand, the *b* value was 11.4 times higher, suggesting improved durability over time (Figure S20). FTIR spectra showed no signs of oxidation even after 8 h UV exposure (Figure S21), though minor smoothing in the fingerprint region after 3 h indicated partial chain breakdown by hydroxyl radicals. pH monitoring revealed minimal acidification compared to DIB/sulfur polymers (Figure S22), and SEM images revealed no visible morphological changes after UV irradiation (Figure S23), further supporting enhanced UV stability.

**Figure 10:**
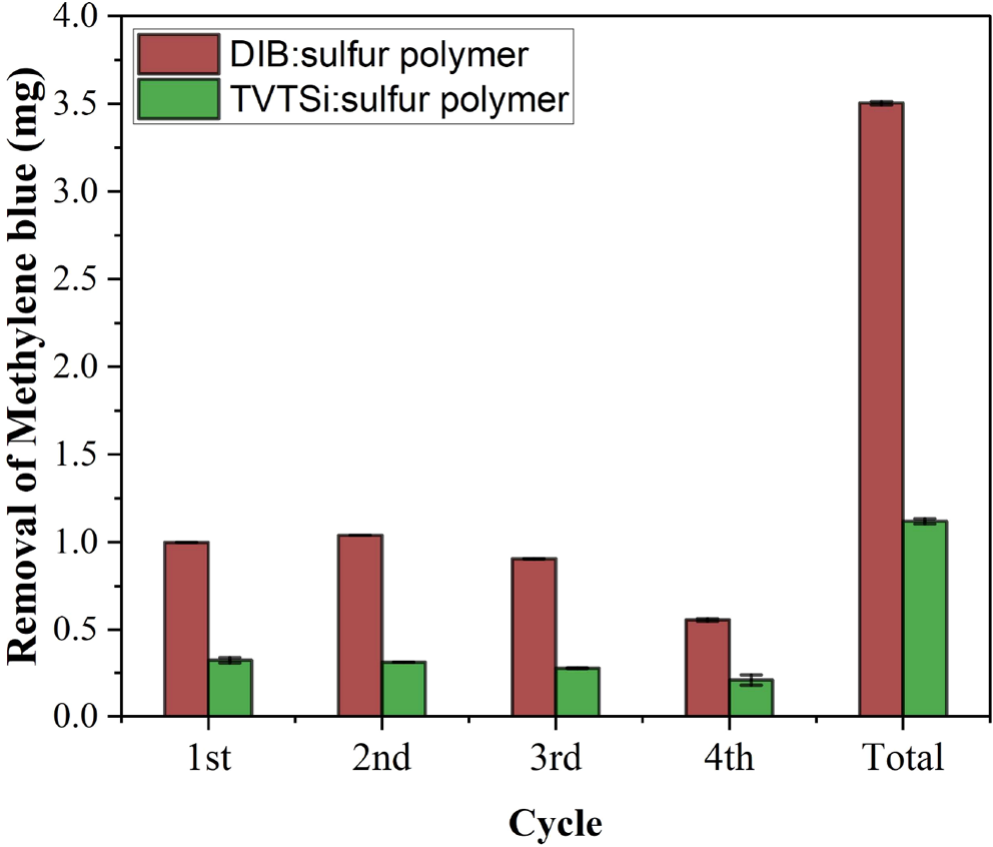
Removal of methylene blue over several cycles using 1,3-diisopropenylbenzene:sulfur and 2,4,6,8,-tetramethyl-2,4,6,8-tetravinylcyclotetrasiloxane:sulfur polymers.

These results demonstrate the feasibility of tailoring sulfur polymer composition to delay degradation and extend catalyst lifetime, albeit with a trade-off in photocatalytic performance. Such tunability enables the rational design of sulfur polymers to suit diverse treatment scenarios, favoring either stability or degradability as needed—for instance, maximizing lifespan in continuous-flow systems or promoting faster degradability to reduce risks of secondary pollution. For example, in this study, we demonstrated that incorporating siloxane-based cross-linkers such as TVTSi can improve UV stability, while varying sulfur content can enhance photocatalytic activity. Recently, Diniz et al.^29^ showed that catalyzed inverse vulcanization offers a versatile approach to modify polymer end-properties, providing a promising pathway to fine-tune both photocatalytic activity and degradability. Future work should focus on optimizing this balance and further assessing the environmental impact of polymer breakdown products.

## 4 Conclusion

This study presents the potential of sulfur polymers synthesized via inverse vulcanization as degradable, metal-free photocatalysts for water purification. Photocatalytic performance was shown to depend on polymer composition, with cross-linkers containing fewer unsaturations (e.g., DIB and PER) yielding higher photocatalytic activity, while siloxane-based cross-linkers (e.g., TVTSi) enhanced UV stability and operational lifespan at the expense of activity.

The findings underscore the unique potential of these materials to be designed for specific treatment needs compared to other metal-free photocatalysts (Table S5), balancing activity and durability. This adaptability supports a broader design framework in which the degradation rate, reusability, and catalytic efficiency can be fine-tuned. Enhanced performance in real water matrices and the contribution of hydroxyl radicals underscore their environmental relevance, although the role of embedded sulfur and the exact degradation mechanism requires further investigation. Limitations include a relatively short lifespan for certain formulations (e.g., DIB:sulfur polymers) and the lack of information on degradation byproducts. Addressing these gaps through studies on polymer degradation pathways, toxicity of breakdown products to ensure environmental safety, and strategies to stabilize sulfur bonding environments will be key to advancing their safe application.

Overall, this work demonstrates the feasibility of sulfur polymer chemistry as a viable route for designing sustainable photocatalysts, offering a promising platform for safer and more sustainable water treatment technologies.

## Supplementary Material



## Notes

Figures S1–S23 including NMR and FTIR spectra, SEM micrographs, thermal analysis (TGA, DSC), Tauc plot, kinetic data, pH evolution profiles, and reusability studies; Tables S1–S5 including polymer performance under varying conditions (concentration, loading, temperature), water quality parameters, and comparison with other metal-free photocatalysts (PDF)
