# Supplementary material for: Metal-Free and Degradable Photocatalyst for Water Decontamination: An Innovative Application for High-Sulfur Content Polymers
Source: ACS Omega. 2025 Oct 17;10(42):49744–57. doi: 10.1021/acsomega.5c04577 (PMC12572999; doi:10.1021/acsomega.5c04577)
Supplement: Supplementary file 1 [file ao5c04577_si_001.pdf]

Supplementary information: Metal-free and degradable photocatalyst for water decontamination: an innovative application for high-sulfur content polymers

Vinicius Diniz[1,2*], Brenda Resendiz-Diaz[1], and Colin R. Crick[1*]

[1]School of Engineering and Materials Sciences, Queen Mary University of London, London, E1 4NS, UK.

[2]Institute of Chemistry, University of Campinas, 13083-970 Campinas, Brazil.

*Authors for correspondence: viniciusdiniz994@gmail.com / *c.crick@qmul.ac.uk*

Content:

Figures S1–S8: ^{1}H NMR spectra for all polymers
Figure S9: FTIR spectra for all polymers
Figure S10: Tauc Plot of the 1,3-diisopropenylbenzene:sulfur polymer.
Figure S11: Pseudo-first-order kinetic fitting and statistical data
Figure S12: Removal of methylene blue by different polymer loadings
Figure S13: Removal kinetics of methylene blue in deionized and tap water
Figure S14: Removal kinetics of caffeine by different polymer loadings
Figure S15: SEM micrographs of 1,3-diisopropenylbenzene:sulfur polymer after irradiation
Figure S16: FTIR spectra of 1,3-diisopropenylbenzene:sulfur polymer after irradiation
Figure S17: pH evolution during photodegradation of methylene blue by 1,3-diisopropenylbenzene:sulfur polymer
Figure S18: TGA of 1,3-diisopropenylbenzene:sulfur polymer after irradiation
Figure S19: DSC analysis of 1,3-diisopropenylbenzene:sulfur polymer after irradiation
Figure S20: Reusability of 2,4,6,8-tetramethyl-2,4,6,8-tetravinylcyclotetrasiloxane:sulfur polymer
Figure S21: FTIR spectra of 2,4,6,8-tetramethyl-2,4,6,8-tetravinylcyclotetrasiloxane:sulfur polymer after irradiation
Figure S22: pH evolution during photodegradation of methylene blue by 2,4,6,8-tetramethyl-2,4,6,8-tetravinylcyclotetrasiloxane:sulfur polymer
Figure S23: SEM micrographs of 2,4,6,8-tetramethyl-2,4,6,8-tetravinylcyclotetrasiloxane:sulfur polymer after irradiation
Table S1: 1,3-diisopropenylbenzene:sulfur polymer performance under different initial methylene blue concentrations
Table S2: 1,3-diisopropenylbenzene:sulfur polymer performance under different polymer loading
Table S3: 1,3-diisopropenylbenzene:sulfur polymer performance under different temperature
Table S4: Tap water quality parameters
Table S5: Comparison of 1,3-diisopropenylbenzene:sulfur polymer performance with other metal-free photocatalysts

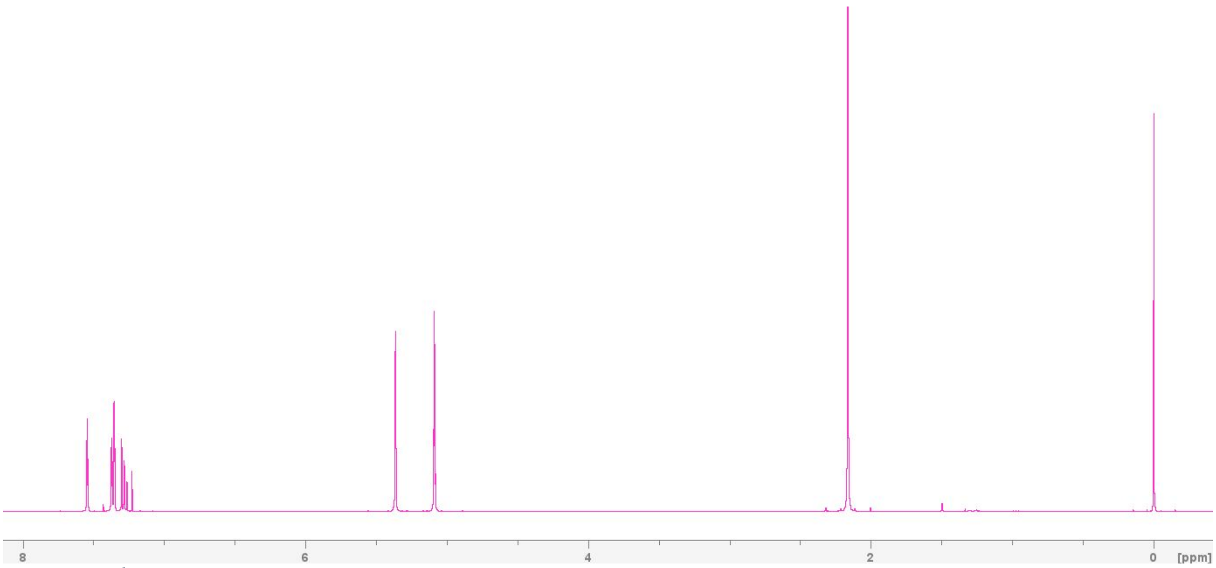

Figure S 1: ¹H-NMR spectrum of the 1,3-diisopropenylbenzene

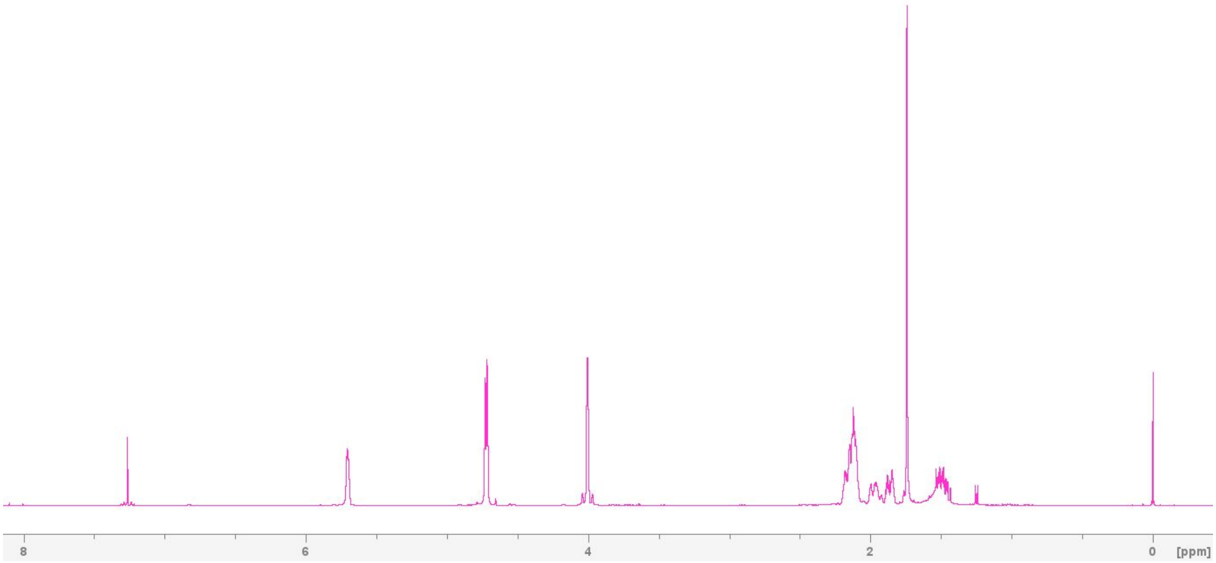

Figure S 2: ¹H_NMR spectrum of the perillyl alcohol.

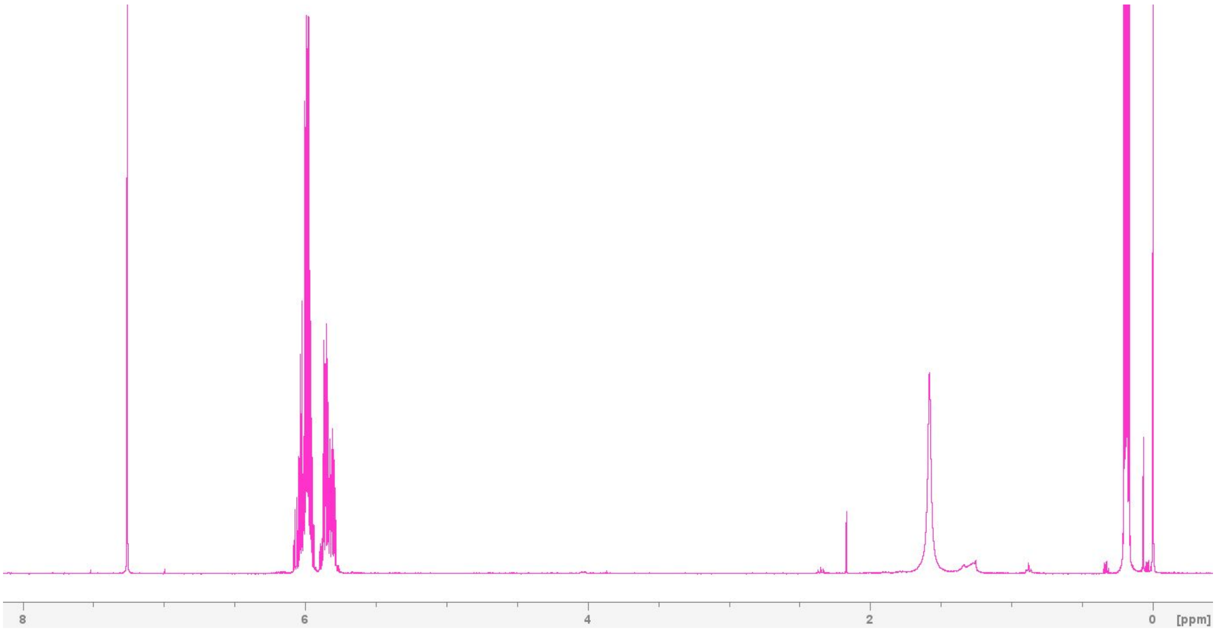

Figure S 3: ^{1}H_NMR spectrum of the 2,4,6,8-tetramethyl-2,4,6,8-tetravinylcyclotetrasiloxane.

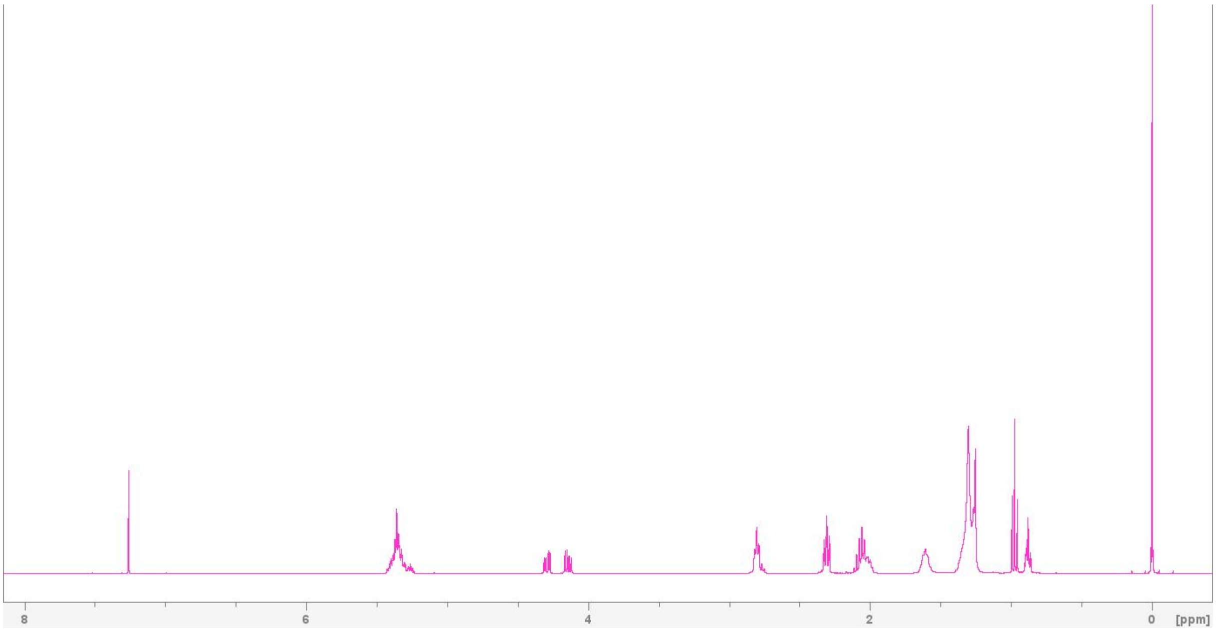

Figure S 4: ^{1}H_NMR spectrum of the linseed oil.

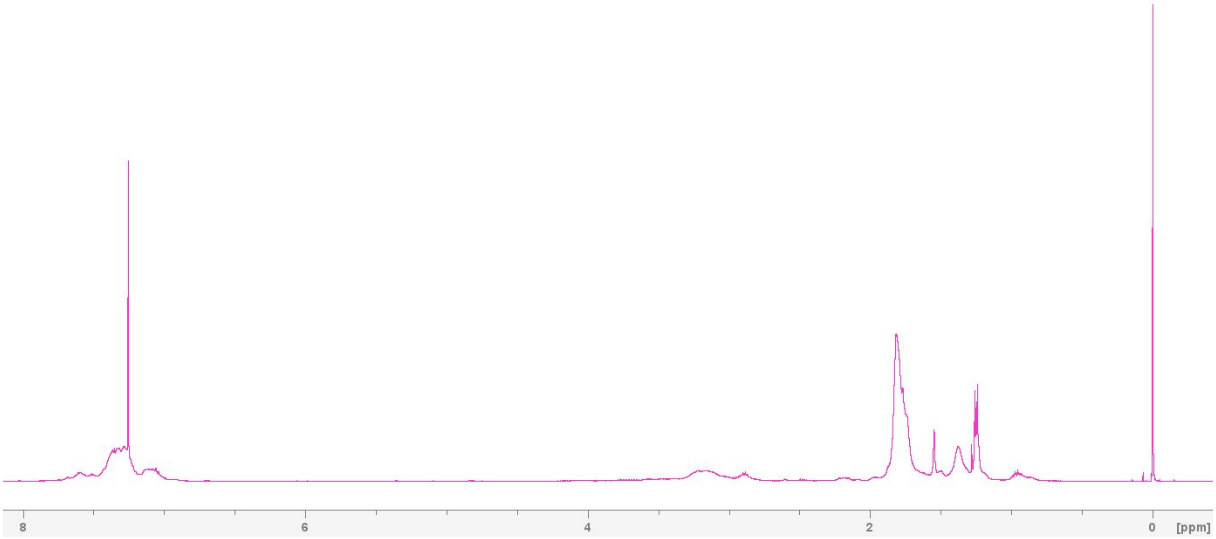

Figure S 5: 1H_NMR spectrum of 1,3-diisopropenylbenzene:sulfur polymer.

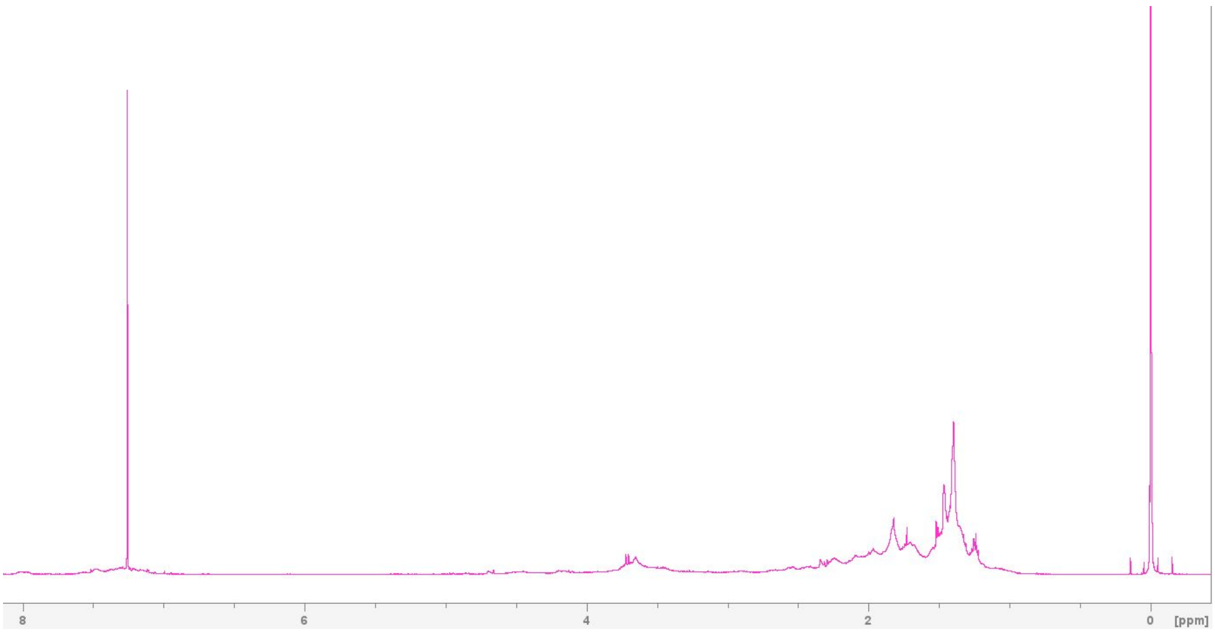

Figure S 6: 1H_NMR spectrum of perillyl alcohol:sulfur polymer.

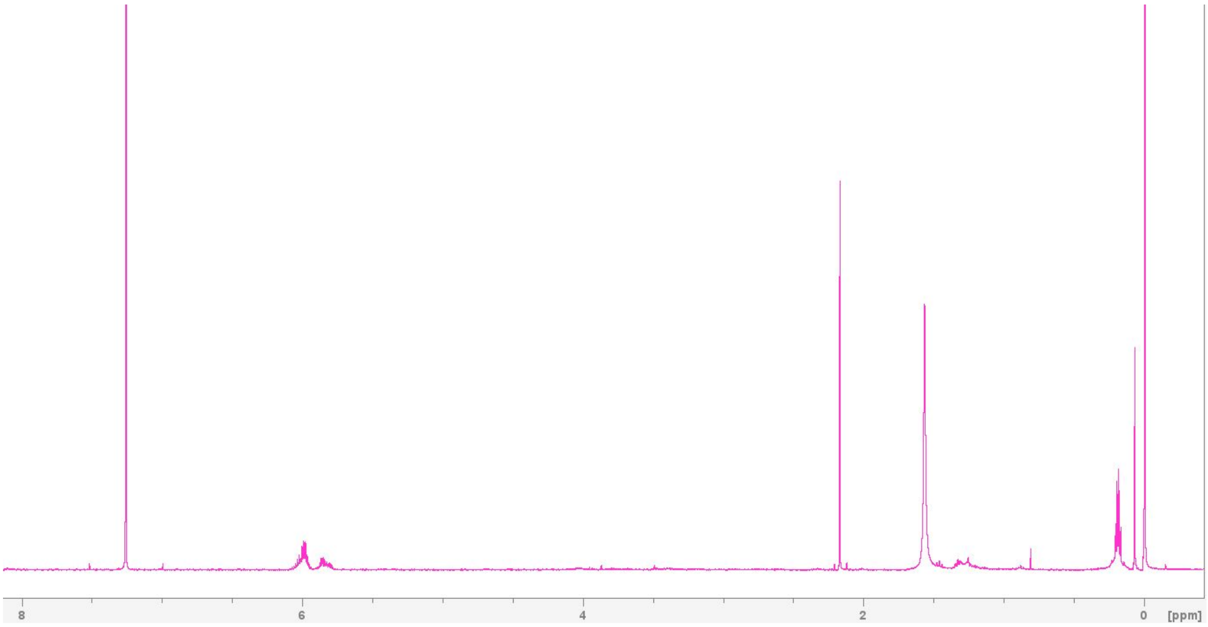

Figure S 7: ^{1}H_NMR spectrum of 2,4,6,8-tetramethyl-2,4,6,8-tetravinylcyclotetrasiloxane:sulfur polymer.

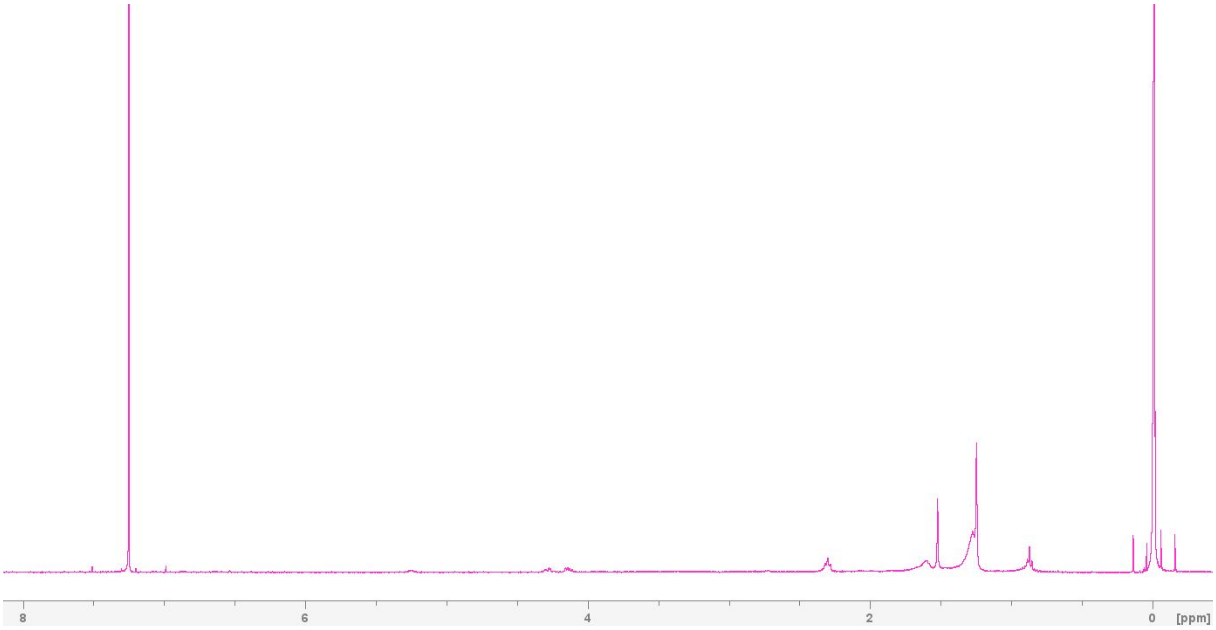

Figure S 8: ^{1}H_NMR spectrum of linseed oil:sulfur polymer.

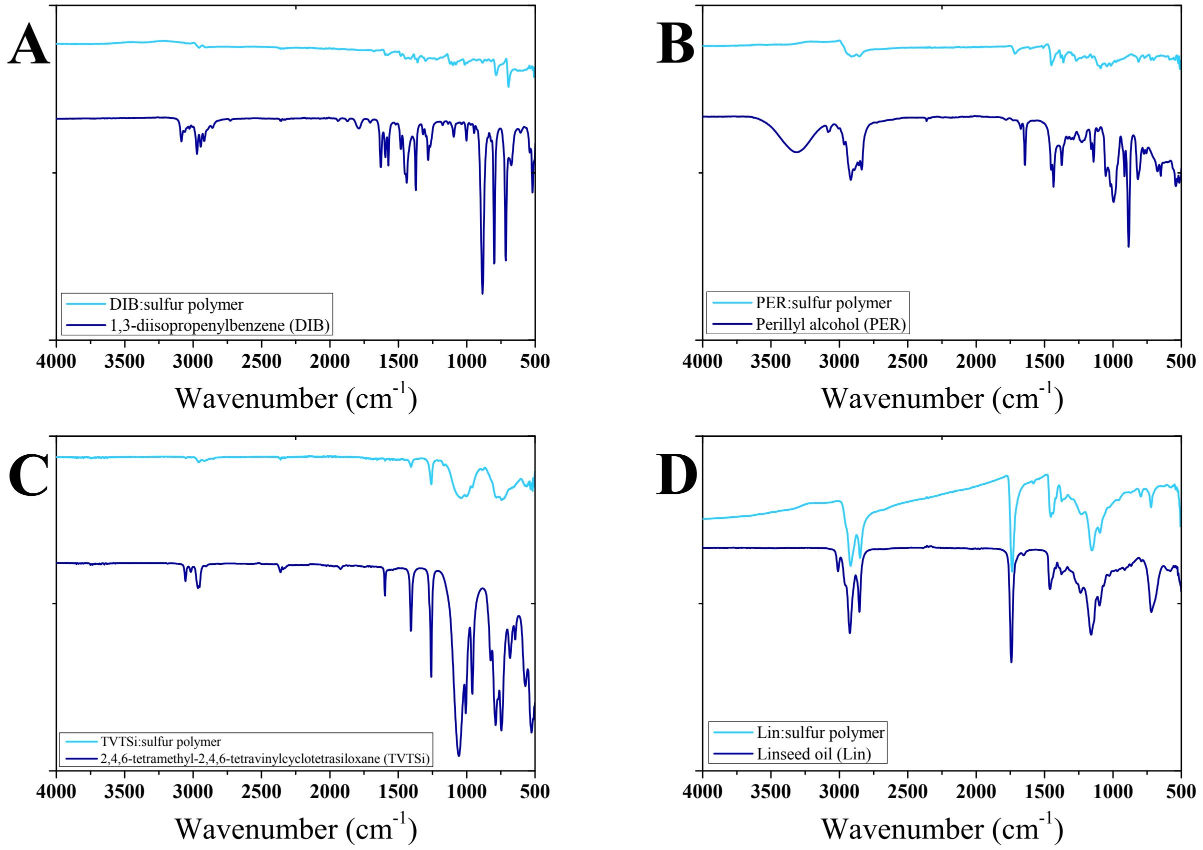

Figure S 9: FTIR spectra of the crosslinkers and sulfur polymer. (A) 1,3-diisopropenylbenzene, (B) Perillyl alcohol, (C) 2,4,6,8-tetramethyl-2,4,6,8-tetravinilciclotetrassiloxane, (D) Linseed oil.

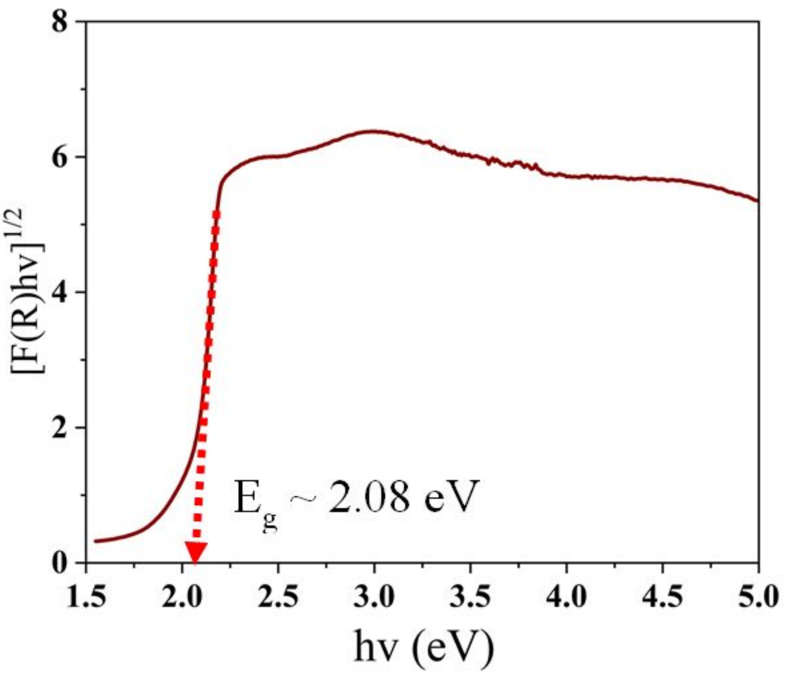

Figure S 10: Tauc Plot of the 1,3-diisopropenylbenzene:sulfur Polymer.

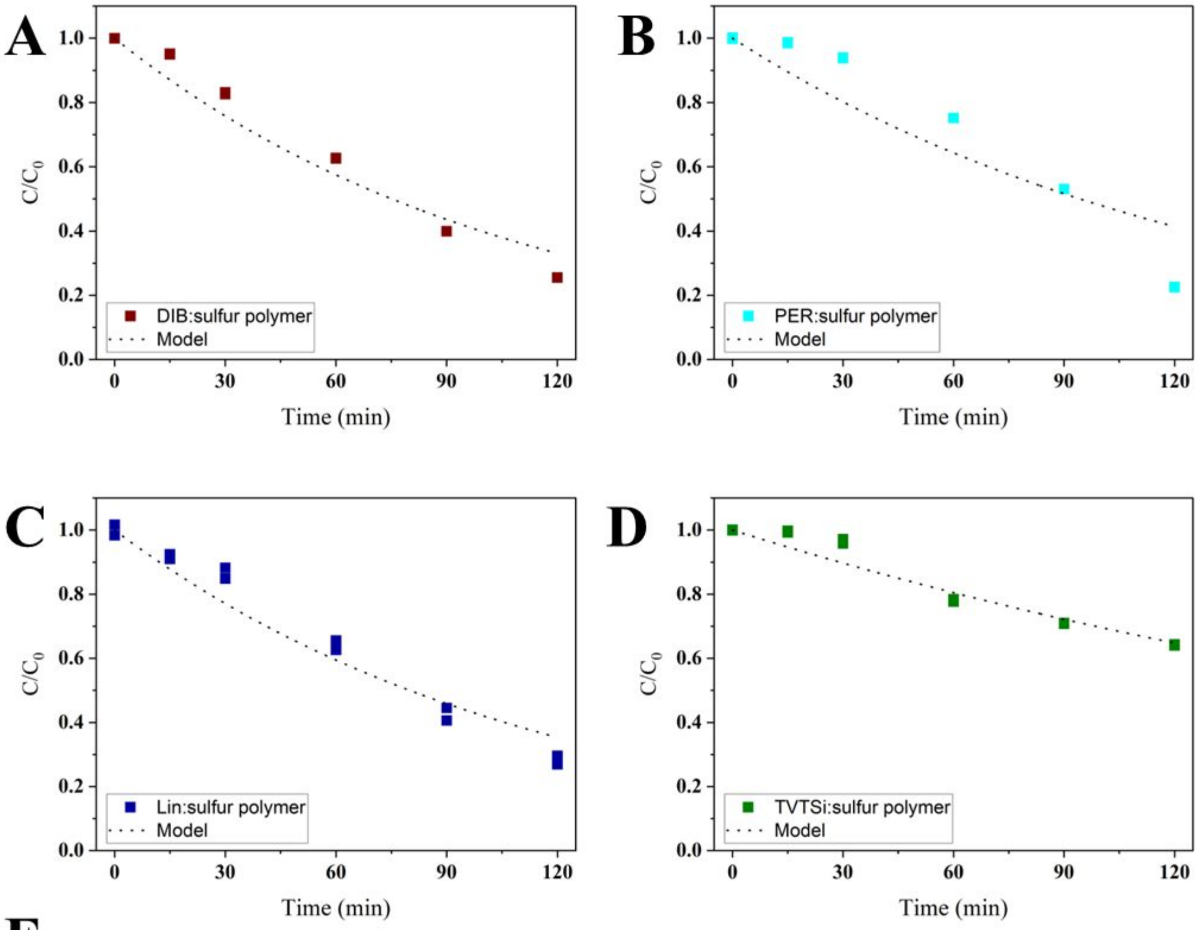

E

Model	First Order Kinetic Model			
Equation	exp(-k*x)			
Plot	DIB:sulfur polymer	Lin:sulfur polymer	PER:sulfur polymer	TVTSi:sulfur polymer
k	0.00923 ± 6.21608E-4	0.00867 ± 5.78008E-4	0.00735 ± 9.92424E-4	0.00362 ± 2.29129E-4
Reduced Chi-Sqr	0.00384	0.00365	0.0135	0.00142
R-Square (COD)	0.95409	0.95201	0.84403	0.93766
Adj. R-Square	0.95409	0.95201	0.84403	0.93766

Figure S 11: Removal capacity of methylene blue by sulfur polymers synthesized with various crosslinkers; (A) DIB: 1,3-diisopropenylbenzene; (B) PER: Perillyl alcohol; (C) TVTSi: 2,4,6,8-tetramethyl-2,4,6,8-tetravinylcyclotetrasiloxane; and (D) Lin: linseed oil, in addition to (E) the pseudo-first-order kinetics models for each polymer.

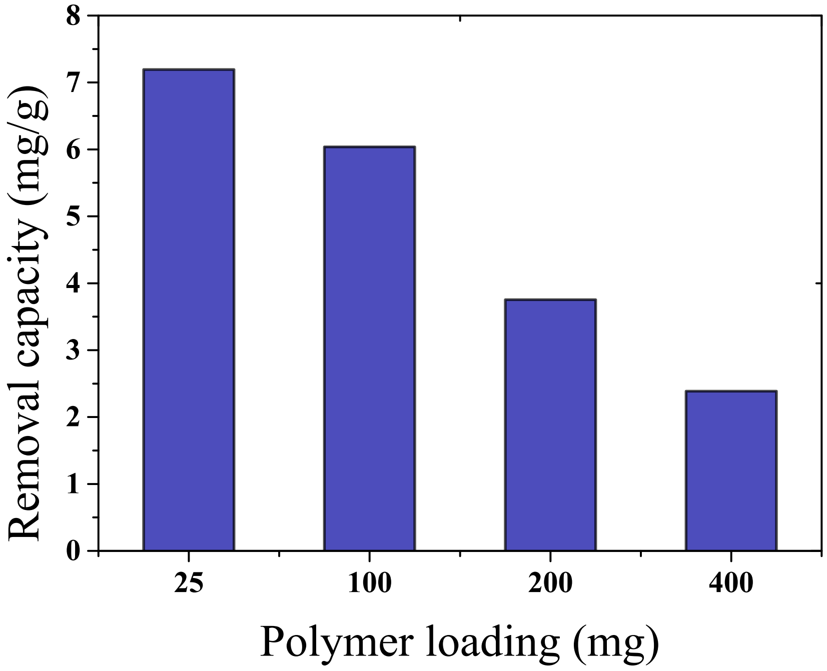

Figure S 12: Removal capacity of methylene blue by the 1,3-diisopropenylbenzene polymer at different polymer loadings.

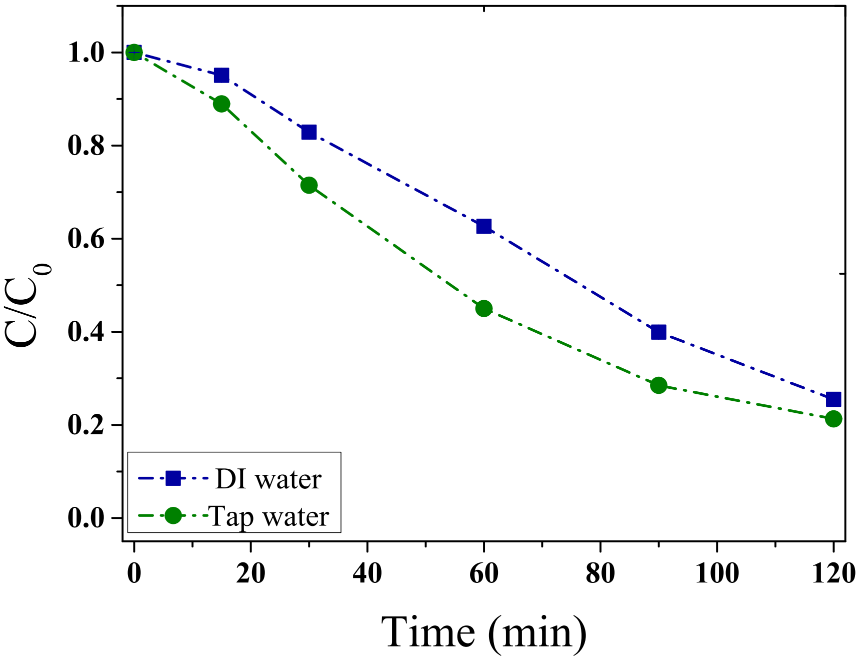

Figure S 13: Degradation of methylene blue by 1,3-diisopropenylbenzene: sulfur polymer in deionized water (DI) and tap water. 200 mg of polymer in 100 mL of 10 mg/L methylene blue solution.

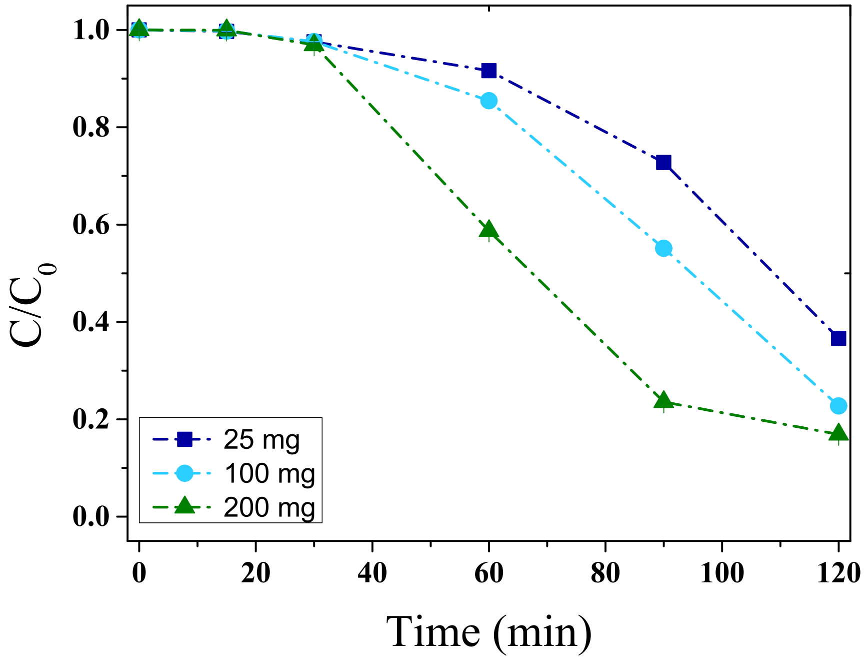

Figure S 14: Degradation of caffeine 1,3-diisopropenylbenzene: sulfur polymer in deionized water. 200 mg of polymer in 100 mL of 20 mg/L caffeine solution.

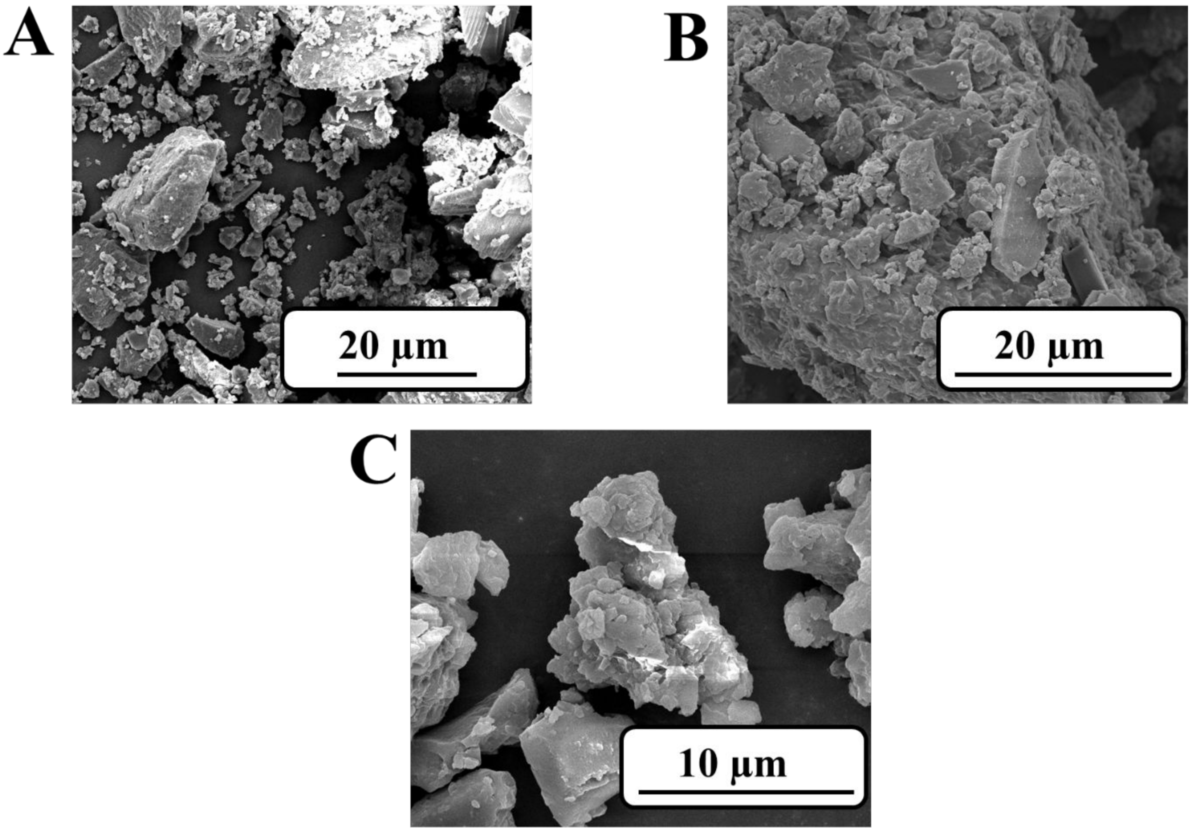

Figure S 15: SEM micrographs of 1,3-diisopropenylbezene:sulfur polymer after 8h of UV irradiation. (A) 20 µm, (B) 20 µm, and (C) 10 µm.

Table S1: Efficiency of DIB:sulfur polymer under different initial MB concentrations (mass of polymer 200 mg, volume 100 mL, and temperature 25 °C)

Initial MB Concentration (mg/L)	q (mg/g)	Removal (%)	K (1/min)
2.5	1.6	>99.9%	0.018
5.0	2.3	89.5	0.012
10.0	3.8	74.5	0.009
15.0	4.2	55.6	0.006

Table S2: Efficiency of DIB:sulfur polymer under different polymer loading (initial MB concentration: 10 mg/L, volume 100 mL, and temperature 25 °C)

Mass of polymer (mg)	q (mg/g)	Removal (%)	K (1/min)
25	7.2	17.0	0.001
100	6.0	56.9	0.006
200	3.8	74.5	0.009
400	2.4	93.4	0.012

Table S3: Efficiency of DIB:sulfur polymer under different temperature (initial MB concentration: 10 mg/L, volume 100 mL, and polymer loading: 200 mg)

Temperature (°C)	q (mg/g)	Removal (%)	K (1/min)
25	3.8	74.5	0.009
35	4.2	71.6	0.009
45	4.2	70.9	0.010
55	2.8	44.6	0.005

Table S 4: Water quality parameter of the Globe Town zone[a] where Queen Mary University London is located.

PARAMETER	UNIT	MEAN
**COLOR**	Pt/Co scale	1.8
**CONDUCTIVITY AT 20 ºC**	µS/cm	618
**PH**	-	7.77
**TURBIDITY**	FTU	0.10
**AMMONIUM AS NH_4**	mg/L	0.19
**CHLORIDE AS CL**	mg/L	54
**CHLORINE (RESIDUAL)**	mg/L	0.80
**FLUORIDE AS F**	mg/L	0.16
**HARDNESS (TOTAL) AS $CACO_3$**	mg/L	262
**NITRATE AS NO_3**	mg/L	29.9
**IRON AS FE**	mg/L	7
**SULFATE AS SO_4**	mg/L	54
**TOTAL ORGANIC CARBON AS C**	mg/L	2.6
**TOTAL PESTICIDES**	µg/L	0.00

a: https://www.thameswater.co.uk/help/water-and-waste-help/water-quality/check-your-water-quality#/results/E14NS

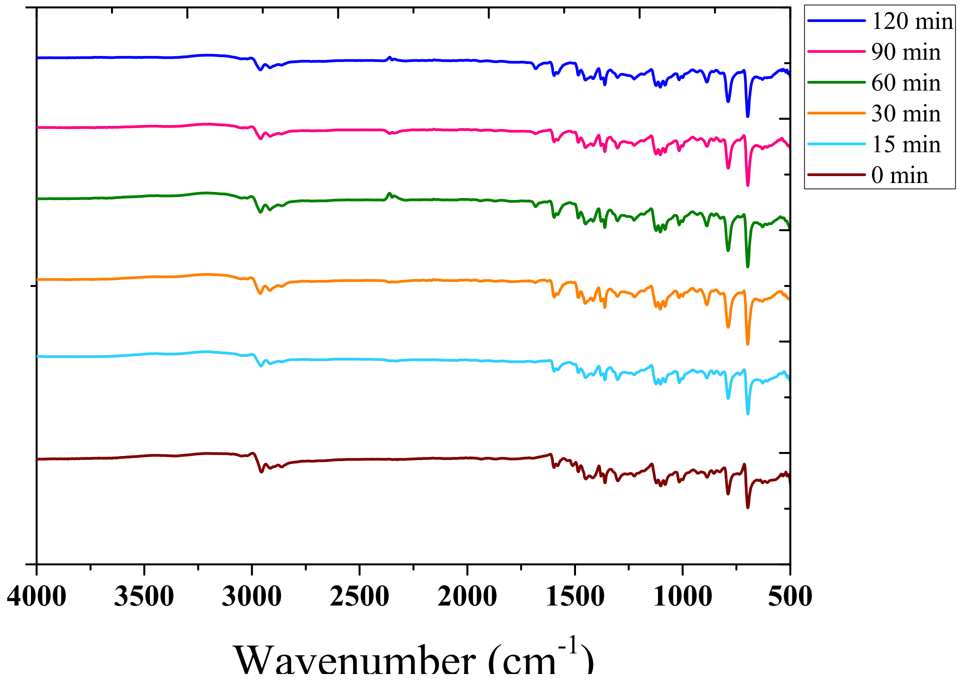

Figure S 16: FTIR spectral progression over the first 120 minutes of UV irradiation on 1,3-diisopropenylbenzene:sulfur polymer.

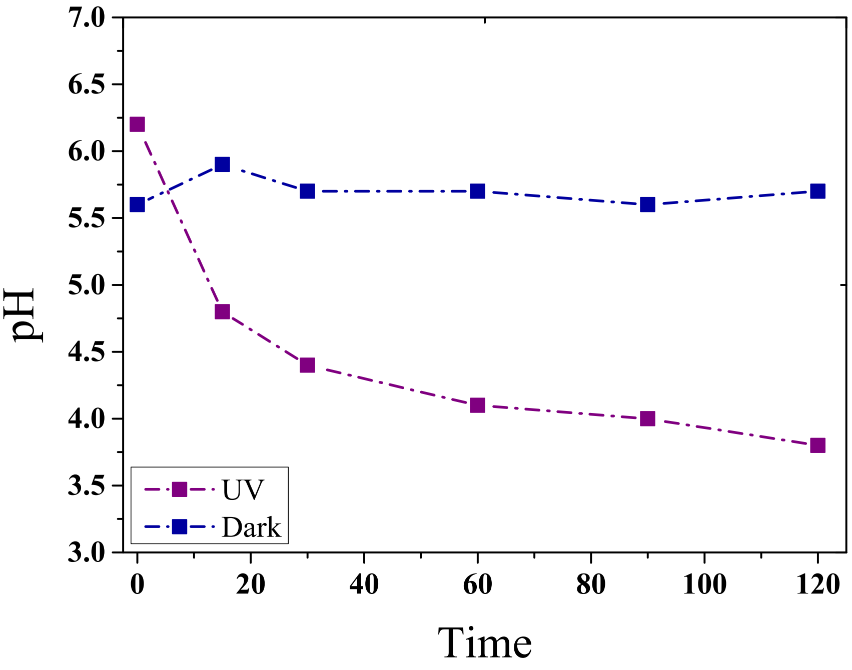

Figure S 17: pH evolution through 1,3-diisopropenylbenzene:sulfur polymer exposed to UV irradiation.

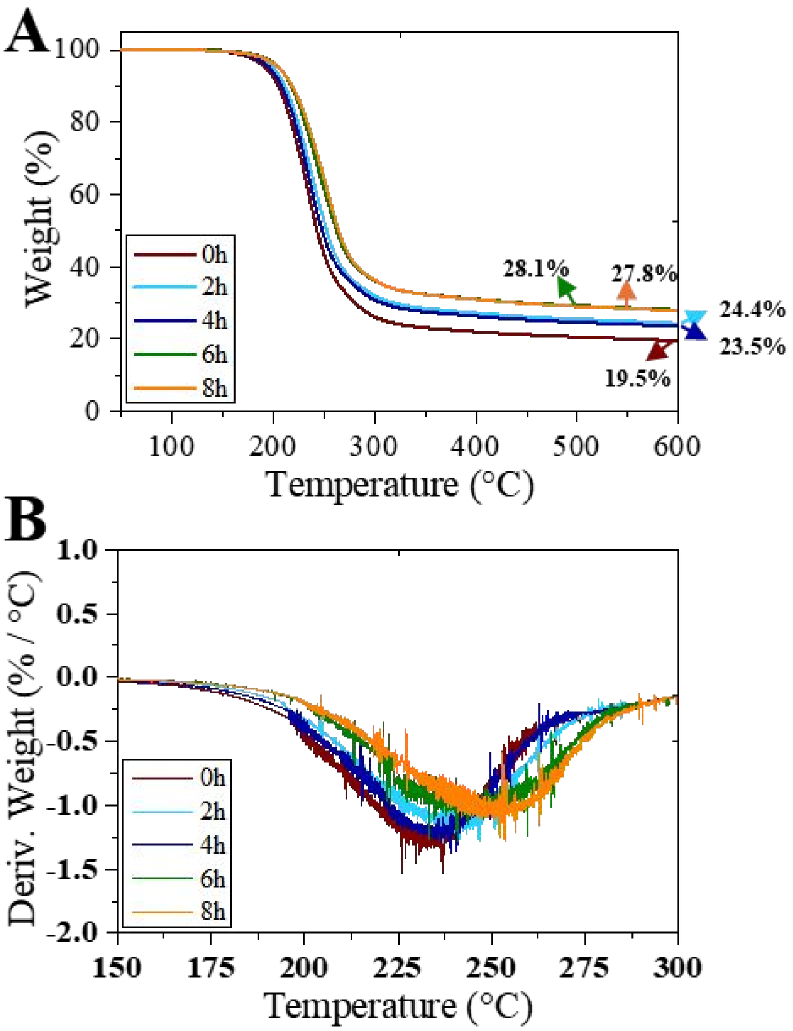

Figure S 18: Thermogravimetric analysis (A) and DTG (B) evolution of 1,3-diisopropenylbenzene:sulfur polymer exposed to UV irradiation.

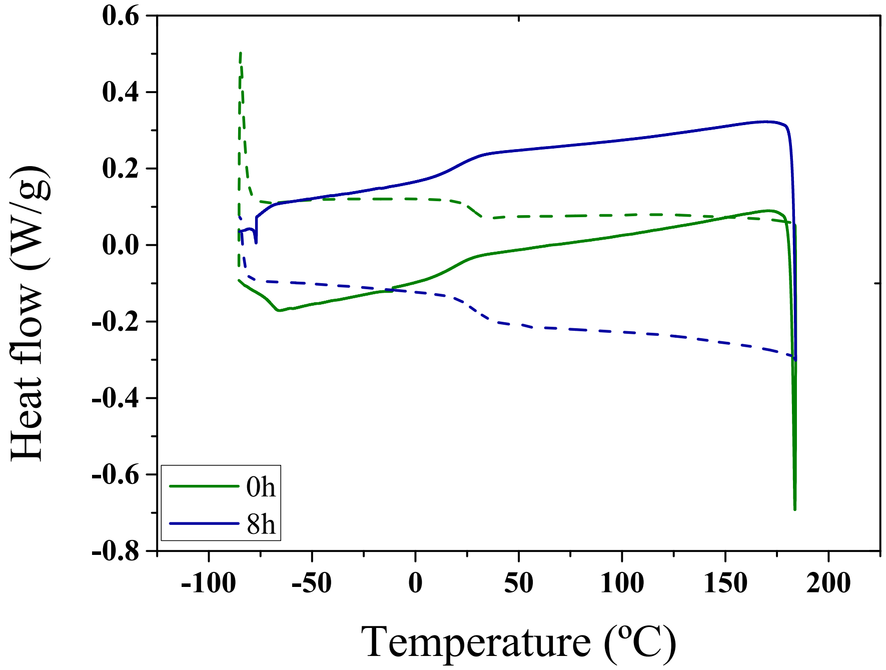

Figure S 19: DSC thermogram evolution of 1,3-diisopropenylbenzene:sulfur polymer exposed to UV irradiation.

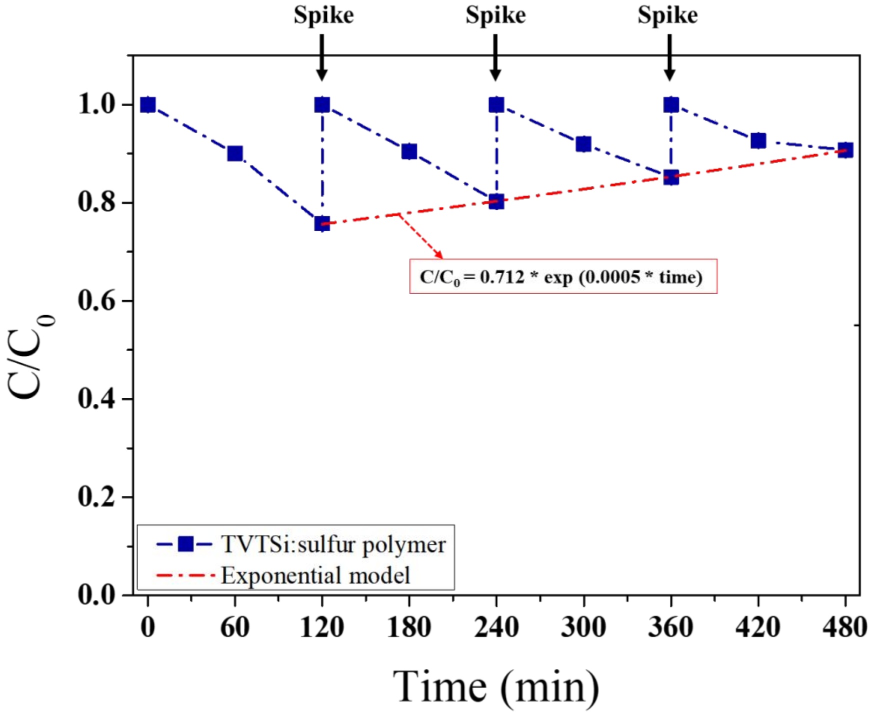

Figure S 20: Reusability of 2,4,6,8-tetramethyl-2,4,6,8-tetravinylcyclotetrasiloxane:sulfur polymer over four photodegradation cycles. The red dash-dotted line represents an exponential model used to predict the removal efficiency at the end of each cycle.

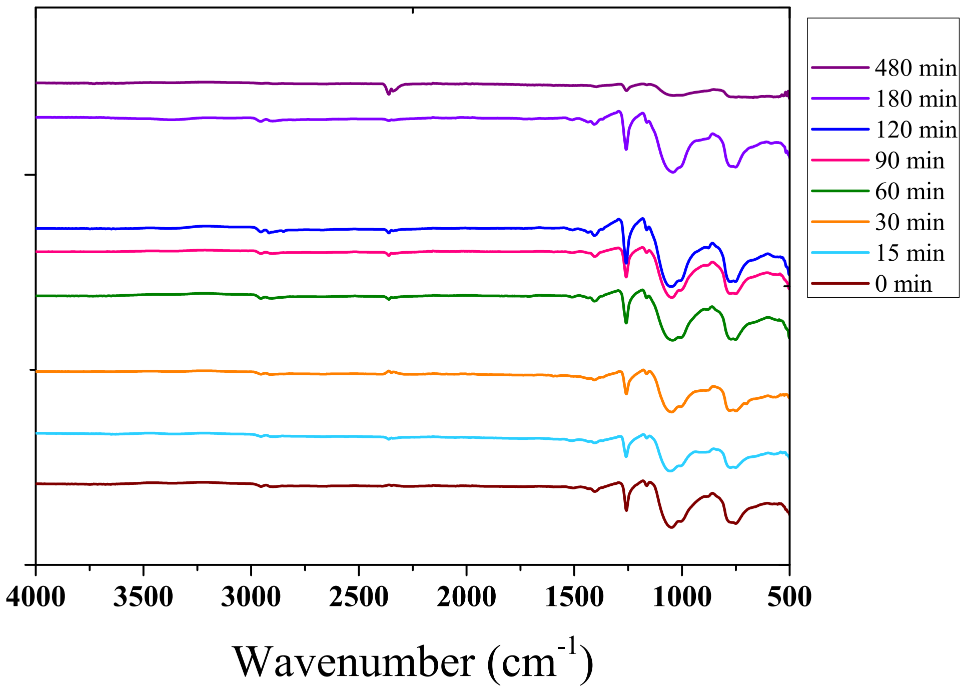

Figure S 21: FTIR spectra of 2,4,6,8-tetramethyl-2,4,6,8-tetravinylcyclotetrasiloxane:sulfur polymer exposed to UV irradiation.

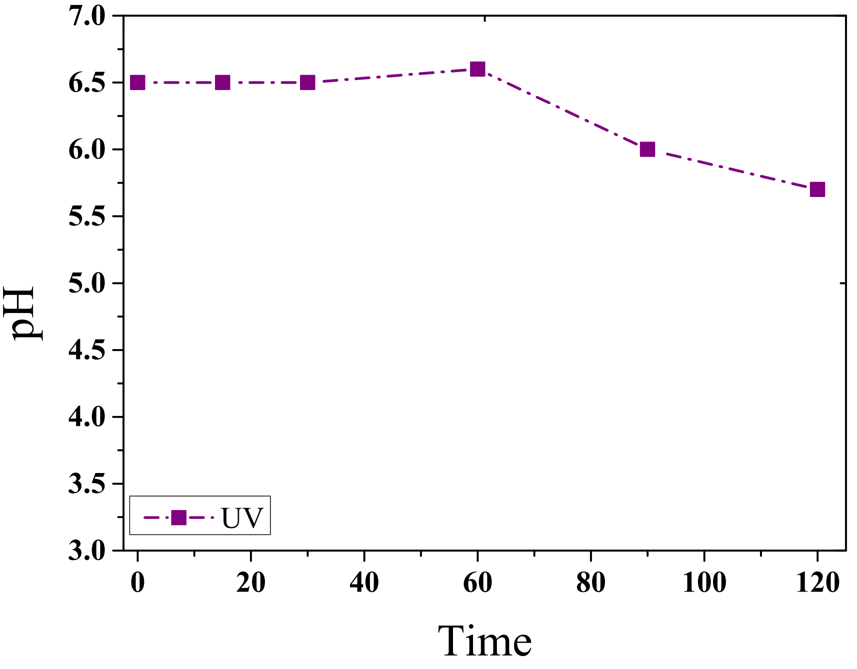

Figure S 22: pH evolution through 2,4,6,8-tetramethyl-2,4,6,8-tetravinylcyclotetrasiloxane:sulfur polymer exposed to UV irradiation.

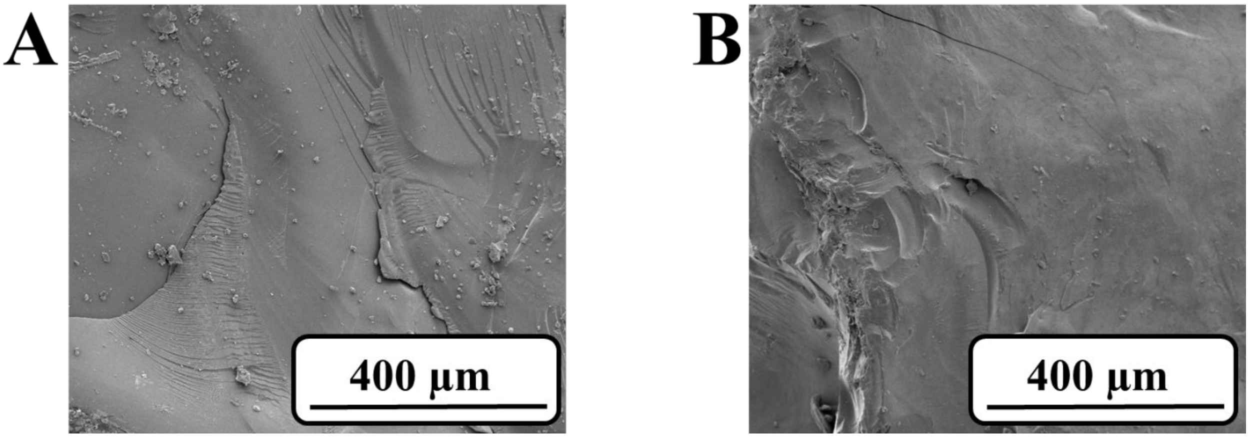

Figure S 23: SEM micrographs of 2,4,6,8-tetramethyl-2,4,6,8-tetravinylcyclotetrasiloxane:sulfur polymer before (A) and after (B) 8h of UV irradiation.

Table S5: Catalyst performance comparison.

Catalyst	Catalyst (mg/L)	C0 (mg/L)	Time (min)	Light source (Watt)	Removal (%)	Ref
Poly(DIB-S)	2000	10	120	UV-C (25 W)	93.4	Present work
rGO-125	200	50	180	Visible (100 W)	>95	1
HAp/AGCN (HAp50/AGCN50)	1500	10	60	Visible (140 W)	93%	2
GO/ZTO/TO	200	20	120	UV-C (60 W)	>95%	3
GO 3wt%	NI	30	60	Visible (500 W)	82.3%	4

[1]10.1016/j.heliyon.2024.e3170; [2]10.1016/j.envres.2025.121582; [3]10.3390/ijms25084367; [4]10.1016/j.envpol.2024.123556
